# Crystal Structure of Chitinase ChiW from *Paenibacillus* sp. str. FPU-7 Reveals a Novel Type of Bacterial Cell-Surface-Expressed Multi-Modular Enzyme Machinery

**DOI:** 10.1371/journal.pone.0167310

**Published:** 2016-12-01

**Authors:** Takafumi Itoh, Takao Hibi, Fumiko Suzuki, Ikumi Sugimoto, Akihiro Fujiwara, Koji Inaka, Hiroaki Tanaka, Kazunori Ohta, Yutaka Fujii, Akira Taketo, Hisashi Kimoto

**Affiliations:** 1 Department of Bioscience, Fukui Prefectural University, Yoshida-gun, Fukui, Japan; 2 Maruwa Foods and Biosciences Inc., Yamatokoriyama, Nara, Japan; 3 Confocal Science Inc., Chiyoda-ku, Tokyo, Japan; 4 Japan Aerospace Exploration Agency, Tsukuba, Ibaraki, Japan; 5 Department of Molecular Biology and Chemistry, Faculty of Medicine, University of Fukui, Yoshida-gun, Fukui, Japan; 6 Department of Environmental and Biotechnological Frontier Engineering, Fukui University of Technology, Fukui, Fukui, Japan; Monash University, AUSTRALIA

## Abstract

The Gram-positive bacterium *Paenibacillus* sp. str. FPU-7 effectively hydrolyzes chitin by using a number of chitinases. A unique chitinase with two catalytic domains, ChiW, is expressed on the cell surface of this bacterium and has high activity towards various chitins, even crystalline chitin. Here, the crystal structure of ChiW at 2.1 Å resolution is presented and describes how the enzyme degrades chitin on the bacterial cell surface. The crystal structure revealed a unique multi-modular architecture composed of six domains to function efficiently on the cell surface: a right-handed β-helix domain (carbohydrate-binding module family 54, CBM-54), a Gly-Ser-rich loop, 1st immunoglobulin-like (Ig-like) fold domain, 1st β/α-barrel catalytic domain (glycoside hydrolase family 18, GH-18), 2nd Ig-like fold domain and 2nd β/α-barrel catalytic domain (GH-18). The structure of the CBM-54, flexibly linked to the catalytic region of ChiW, is described here for the first time. It is similar to those of carbohydrate lyases but displayed no detectable carbohydrate degradation activities. The CBM-54 of ChiW bound to cell wall polysaccharides, such as chin, chitosan, β-1,3-glucan, xylan and cellulose. The structural and biochemical data obtained here also indicated that the enzyme has deep and short active site clefts with endo-acting character. The affinity of CBM-54 towards cell wall polysaccharides and the degradation pattern of the catalytic domains may help to efficiently decompose the cell wall chitin through the contact surface. Furthermore, we clarify that other Gram-positive bacteria possess similar cell-surface-expressed multi-modular enzymes for cell wall polysaccharide degradation.

## Introduction

Structural polysaccharides, such as cellulose and chitin, are the most abundant biomass resource on earth, and are widely distributed in plants, fungi, insects and crustaceans. These polysaccharides have attracted much attention as potential renewable sources of energy, fuels and functional materials. For example, cellulose of plant cell walls, a linear polymer of D-glucose with the β-1,4-linkage, can be converted into ethanol biofuel via fermentation [1]. Another major biomass resource, chitin, composed of *N*-acetyl-D-glucosamine (GlcNAc) as a repeating unit with the β-1,4-linkage, and chitin-derived sugars, e.g., chitosan, oligo- and monosaccharides, have beneficial effects as elicitors and anti-tumor agents. Accordingly, chitin is of industrial, agricultural, cosmetic and medicinal interest [2–4]. Although such structural polysaccharides are profitable, the conversion processes of these structural polysaccharides are limited because of their tightly packed structures. Raw polysaccharide materials are hydrolyzed with concentrated HCl or H_2_SO_4_ in manufacturing processes, which are an environmental burden and operational risk. On the other hand, particular bacteria can efficiently degrade and use these recalcitrant polysaccharides as an energy source by employing a large number of strategies [2, 5]. Secreted glycoside hydrolases that target structural polysaccharides, e.g., cellulases and chitinases, play important roles in such degradation strategies. These enzymes often contain carbohydrate-binding modules that bind to the polysaccharides of target solid surfaces and aid depolymerization [6, 7]. Copper-dependent redox enzymes, called lytic polysaccharide monooxygenases, were recently discovered [8, 9]. These enzymes catalyze oxidative cleavage of polymer chains on flat surfaces, make multiple nicks and assist other glycoside hydrolases in attacking the polymer chains. Furthermore, some Gram-positive cellulolytic bacteria, e.g., *Acetivibrio cellulolyticus* and *Ruminiclostridium cellulolyticum* (formerly known as *Clostridium cellulolyticum*), produce a concerted and multi-functional “cellulosome” enzyme complex that functions to degrade plant cellulose efficiently [10]. Displaying and concentrating enzymes on bacterial cell surfaces is likely to effectively facilitate the transport of the hydrolyzed products into the cell before they diffuse away from the cell surface.

**Fig 1:**
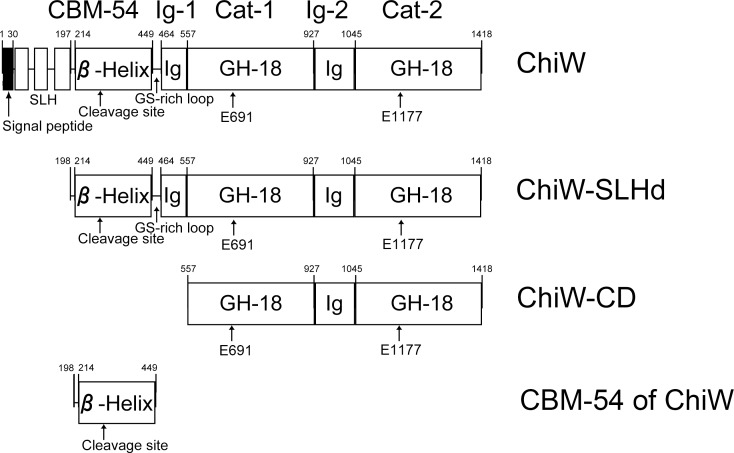
Schematic representations of the primary structures of ChiW proteins. ChiW denotes the full-length protein of *Paenibacillus* sp. str. FPU-7 ChiW; ChiW-SLHd (CBM-54, Ig-1, Cat-1, Ig-2 and Cat-2 domains), ChiW-CD (Cat-1, Ig-2 and Cat-2 domains) and CBM-54 of ChiW (CBM-54 domain) denote the truncated versions of ChiW. Boxes denote annotated structural elements. The numbers indicate the amino acid residue numbers. ChiW is specifically cleaved between Asn282 and Ser283. The position is pointed out as the cleavage site. Glu691 and Glu1177 are predicted to be catalytic residues and act as generic acids.

*Paenibacillus* sp. str. FPU-7 (*P*. str. FPU-7) has been isolated from soil and degrades crystalline chitin readily [11]. Genomic and biochemical analyses of the FPU-7 strain have revealed that the bacterium secretes at least seven chitinases, one of which is a unique high-molecular-mass (150 kDa) chitinase, termed ChiW. This enzyme has three surface-layer homology (SLH) domains (~18 kDa) (Fig 1); it is specifically expressed on the surface of the bacterial cell and degrades chitin [11, 12]. In general, the SLH domains are composed of three repeats of highly conserved sequences and bind noncovalently to glycan backbones of the peptidoglycan of Gram-positive bacteria, whereupon the cell wall is surrounded by the congregated proteins with SLH domains as a cell envelope or surface layer [13]. We propose that cell-surface-expressed enzymes can be used to enhance polymer degradation [11]. Based on comparative sequence analyses, ChiW has two glycoside hydrolase family 18 (GH-18) chitinase catalytic domains (~42 kDa each; Fig 1) and one carbohydrate-binding module family 54 (CBM-54) (~25 kDa; Fig 1), as classified in the Carbohydrate-Active enZYmes (CAZy) database [14]. No typical chitin-binding module can be identified [11]. The structures and functions of the remaining regions (a total of 23 kDa) of ChiW remain unknown.

In this study, we have determined the crystal structure of the bacterial cell-surface enzyme ChiW and demonstrated that this elaborate monomeric enzyme is composed of six distinct structural domains. The protein fold of CBM-54 determined here is the first structural fold in this CBM family and is similar to those of carbohydrate lyases. However, the CBM-54 of ChiW showed binding capacity towards various insoluble polysaccharides rather than degradation activity. Structure motif mining indicates that such peculiar multi-modular biological devices are common in Gram-positive bacteria. This unique multi-functional and multi-modular enzyme provides useful functional information regarding the bacterial cell envelope and provides insights into bacterial efficient strategies for biodegradation of structural polysaccharides.

## Materials and Methods

### Chemicals and reagents

All chemicals and reagents were analytical-grade and purchased from Wako Pure Chemical (Osaka, Japan) or Sigma-Aldrich (St. Louis, MO, USA), unless otherwise stated.

### Production and purification of ChiW-SLHd, ChiW-CD and CBM-54 of ChiW

ChiW-SLHd (Val198 to Lys1418), ChiW-CD (Val557 to Lys1418), ChiW-SLHd-DM (active site residues, Glu691 and Glu1177 substituted to Gln; E691Q and E1177Q) and ChiW-CD-DM (E691Q and E1177Q) of *P*. str. FPU-7 (Fig 1) were overexpressed in *Escherichia coli* (*E*. *coli*) and purified as described previously [12]. The expression vector for the recombinant protein composed of CBM-54 of ChiW (Val198 to Phe449) was prepared by DNA truncation from the ChiW-SLHd expression vector with the KOD-Plus-Mutagenesis Kit (Toyobo, Osaka, Japan). The following primers were used: 5′-CATCACCATCACCATCACTAAGGATCCG-3′ and 5′-GAAGCTCCCGCCTGTCGTCACGGAG-3′. The CBM-54 of ChiW was also overexpressed in *E*. coli BL21 and purified from the cell extract using a procedure similar to that used for ChiW-SLHd [12]. Solutions containing these proteins were dialyzed at 4°C overnight against 10 mM HEPES buffer (pH 7.0) and used as a purified protein source. The protein concentrations were determined using the Bio-Rad Protein Assay Kit (Hercules, CA, USA) based on the Bradford method [15], with bovine serum albumin as the standard or were determined by UV spectrophotometry using the molar extinction coefficients, *ε*_280_ = 215,374 (M^−1^ cm^−1^) for ChiW-SLHd or 193,900 (M^−1^ cm^−1^) for ChiW-CD [12, 16] according to the ExPASy ProtParam tool server (http://web.expasy.org/protparam/) [17]. The protein purities were assessed by sodium dodecyl sulfate polyacrylamide gel electrophoresis (SDS-PAGE) followed by Coomassie Brilliant Blue (CBB) R-250 staining [18].

### Crystallization and X-ray diffraction

Crystals of the purified ChiW-CD (10 mg ml^−1^) were prepared by the sitting-drop vapor diffusion method, as described previously [16]. X-ray diffraction images of the ChiW-CD crystal were processed to a resolution of 2.03 Å (Table 1). ChiW-CD was also co-crystallized with the trisaccharide substrate (GlcNAc)_3_ using the same crystallization conditions. The purified ChiW-SLHd was concentrated using an Amicon Ultra-4 concentrator with a 10,000 Da molecular weight cutoff membrane (Millipore, Billerica, MA, USA) to a final concentration of 30 mg ml^−1^. Commercial crystal screening kits from Hampton Research (Alisa Viejo, CA, USA) and Emerald BioSystems (Bainbridge Island, WA, USA) were used for the initial screening of the crystallization conditions at 20°C using the sitting-drop vapor-diffusion method. Initial crystals of ChiW-SLHd were grown from the No. 9 solution of Emerald BioSystems Wizard I random sparse matrix crystallization screen kit containing 1.0 M (NH_4_)_2_HPO_4_ and 0.1 M sodium acetate buffer, pH 4.5. The crystals suitable for X-ray analysis were obtained using the sitting-drop vapor-diffusion or counter-diffusion [19, 20] crystallization methods. The sitting drops were prepared by mixing 3 μl of the enzyme solution with an equal volume of reservoir solution containing 0.8–1.3 M (NH_4_)_2_HPO_4_ and 0.1 M sodium citrate buffer, pH 4.5–5.5, and equilibrated at 20°C with 0.5 ml of the reservoir solution. The counter-diffusion crystallization method was carried out under a microgravity environment in the Japanese Experiment Module “Kibo” at the International Space Station (ISS) [21] with the same crystallization solutions (launch date of ISS: September 26, 2014, return date to Earth: November 10, 2014). The rod-shaped crystals grew to a maximum of 0.1 × 0.1 × 1.0 mm. These single crystals were soaked for 30 s at 20°C in a cryoprotectant solution containing 3.5 M sodium formate, 0.8–1.3 M (NH_4_)_2_HPO_4_ and 0.1 M sodium citrate buffer, pH 4.5–5.5. The crystals were placed in a cold nitrogen gas stream at −173°C. X-ray diffraction images of the crystals were mainly collected using ADSC Quantum 315 CCD X-ray detectors (Poway, CA, USA) with synchrotron radiations (*λ* = 0.98 Å at the BL-17A station of the Photon Factory or *λ* = 1.00 Å at the BL-26B2/38B1 stations of SPring-8). Images were processed using the HKL-2000 program [22] (Table 1).

**Table 1:** Data collection and refinement statistics for ChiW structures.

	ChiW-CD^a^	ChiW-SLHd	ChiW-CD/(GlcNAc)_2_
Space group	*P*2_1_2_1_2_1_	*P*2_1_2_1_2_1_	*P*2_1_2_1_2_1_
Unit cell parameters (Å)	a = 112.0, b = 128.2, c = 162.1	a = 114.3, b = 123.5, c = 130.9	a = 113.0, b = 127.2, c = 161.4
**Data collection**			
Resolution limit (last shell)^b^ (Å)	50.0–2.03 (2.07–2.03)	50.0–2.10 (2.14–2.10)	50.0–2.60 (2.64–2.60)
Measured reflections	1,057,801	618,912	520,794
Unique reflections	149,010 (7,427)	108,304 (5.361)	70,506 (3,476)
Redundancy	7.1 (6.9)	5.7 (5.5)	7.5 (7.4)
Completeness (|*I*|>σ|*I*|) (%)	99.0 (100)	99.9 (100.0)	99.6 (99.2)
*<I*/σ(*I)>*	30.0 (6.6)	30.4 (4.4)	25.3 (5.8)
*R*_merge_ (%)^c^	5.8 (36.3)	4.7 (36.7)	7.3 (38.0)
*R*_pim_ (%)^d^	2.4 (14.9)	2.1 (17.1)	2.9 (14.9)
CC_1/2_ of last shell (%)^e^	93.6	92.6	95.0
Wilson *B* factor (Å^2^)	21.8	22.0	34.7
**Refinement**			
Final model	858 (A) and 859 (B) amino acids, 1333 water molecules, 2 phosphate ions	1203 amino acids, 1358 water molecules, 1 sodium, 1 phosphate ion, 10 formate ions	860 (A) and 859 (B) amino acids, 203 water molecules, 2 phosphate ions, 2 (GlcNAc)_2_ molecules
Resolution limit (Å)	50.0–2.03 (2.08–2.03)	50.0–2.10 (2.16–2.10)	50.0–2.61 (2.68–2.61)
Used reflections	141,163 (10,374)	102,679 (5,370)	66,976 (3,459)
Completeness (|*F*| > σ|*F*|) (%)	98.8 (99.3)	99.7 (98.7)	99.0 (91.9)
Average *B*-factor (Å^2^)			
Protein	29.8 (A), 28.2 (B)	36.2	42.4 (A), 44.7 (B)
Water	37.7	41.1	30.6
(GlcNAc)_2_			34.0 (A), 38.2 (B)
*R*-factor (%)^f^	18.5 (22.5)	17.5 (21.5)	20.5 (27.6)
*R*_free_ (%)^g^	22.6 (27.1)	22.2 (25.7)	24.8 (32.0)
Root-mean-square deviations			
Bond (Å)	0.020	0.022	0.016
Angle (°)	2.000	2.018	1.827
Ramachandran plot (%)			
Favored region	96.7	95.6	92.8
Allowed region	3.2	3.8	6.4
Outlier region	0.2	0.7	0.9

^a^ Data collection statistics for ChiW-CD are taken from previously published data [16].

^b^ Data in the highest resolution shells are given in parentheses.

^c^ *R*_merge_ = Σ_*hkl*_∑_i_|*I*_i_(hkl)–<*I*(hkl)>| / Σ_*hkl*_∑*I*_i_(hkl) × 100, where *I*_i_(hkl) is the intensity of individual reflection and <*I*(hkl)> is the mean intensity of all reflections.

^d^ *R*_pim_ =  Σ_*hkl*_[1/(N−1)]^1/2^Σ_i_|*I*_i_(hkl)−<*I*(hkl)>|/Σ_hkl_ Σ_i_*I*_i_(hkl) × 100, where *I*_i_(hkl) is the intensity of individual reflection and <*I*(hkl)> is the mean intensity of all reflections.

^e^ CC_1/2_ is the correlation coefficient between random half-datasets.

^f^ *R*-factor = ∑|*F*_o_−*F*_c_|/∑|*F*_o_| × 100, where *F*_o_ is the observed structure factor and *F*_c_ is the calculated structure factor.

^g^ *R*_free_ was calculated from 5% of the reflections selected randomly.

### Structure determination and refinement

The initial model of the ChiW-CD crystal structure was obtained using the molecular replacement (MR) method with the PHASER program ver. 2.3 [23] and the *Bacillus* chitinase A1 catalytic domain [24] deposited in the RCSB Protein Data Bank (PDB) [25] (PDB ID: 1ITX). The initial model was then rebuilt using the Buccaneer automated protein model building software [26] from the CCP4 6.2.0 suite [27]. The model was refined and manually rebuilt using Refmac5 ver. 5.8 [28] and Coot ver. 0.8 [29] at 2.03 Å (Table 1). The initial model of ChiW-SLHd was also determined by the MR method and automated protein model building. For this phase determination, the refined ChiW-CD structure was used as the reference model for the MR method. The model was also completed by the Refmac and Coot programs (Table 1). The crystal structure of ChiW-CD complexed with the reaction product (GlcNAc)_2_ was also determined by the MR method and Refmac programs (Table 1). Structural similarity was searched for using the PDB and the DALI program [30]. Structural alignments were conducted by superimposition using a fitting program in Coot. Structural figures were prepared by PyMol (DeLano Scientific, Palo Alto, CA, USA).

### Amino acid sequence analysis

The amino acid sequence of ChiW was divided into seven domains (SLH, CBM-54, GS-rich loop, two immunoglobulin-like (Ig-like) and two catalytic domains) guided by the crystal structure. Amino acid sequence analysis of each domain was performed using BLASTP [31] and ClustalW [32] via the National Library of Medicine. The 74 amino acid sequences of the CBM-54 family in the CAZy database [14] were aligned by ClustalW and the phylogenetic tree of CBM-54 domains was plotted using NJplot with the neighbor-joining (NJ) method [33, 34].

### Measurement of released (GlcNAc)_2_ in the enzymatic reaction

The pH optimum of this enzyme is pH 5.5 [12] and the assays were performed in triplicate at the same pH. The enzyme reactions were conducted at 37°C as follows: the reaction mixture consisted of 5 mM sodium acetate buffer (pH 5.5), 0.5% (w/v) colloidal chitin prepared from powdered α-chitin [12] and 100 nM ChiW-SLHd in a 100 μl reaction volume, or 5 mM sodium acetate buffer (pH 5.5), 2 mM (GlcNAc)_3_ and 100 nM ChiW-CD in a 100 μl reaction volume. The degradation progress was terminated by withdrawing 10 μl aliquots from the reaction solution and then adding 10 μl acetonitrile at 0, 5, 10 and 20 min for α-chitin, or 1, 3, 10 and 20 min for (GlcNAc)_3_. The amount of product (GlcNAc)_2_ in the mixture was analyzed by a TOSOH 8020 HPLC system equipped with a TSKgel Amide-80 column (4.6 × 250 mm; Tosoh Co., Tokyo, Japan). The products were eluted with a mobile phase of 70% (v/v) acetonitrile and detected at 210 nm. One unit of activity was defined as the amount of enzyme catalyzing the production of 1 μmol of product per min.

### Degradation assay and binding experiment of CBM-54 toward insoluble polysaccharides

The following insoluble polysaccharides (Wako Pure Chemical) were used for the assays: powdered chitin, chitosan, β-1,3-glucan, cellulose and xylan. The assays were carried out at least three times. The reducing sugar released from the enzymatic reaction for the insoluble polysaccharides was estimated using the 3,5-dinitrosalicylic acid (DNS) method [35] with 0.1–1.0 mM GlcNAc or glucose as a standard. The reaction mixture consisted of 50 mM sodium acetate buffer (pH 5.5), 5 mg polysaccharide and 10 μM CBM-54 of ChiW in a 1 ml reaction volume. After 1 h incubation at 37°C, 50 μl aliquots from the reaction solution were mixed with 50 μl DNS reagent [35]. The absorbance of the mixtures was recorded at 595 nm.

The binding experiment was conducted by adding 10 μg of the CBM-54 of ChiW to 2 mg of insoluble polysaccharides in 200 μl of 10 mM sodium citrate buffer (pH 5.5). The mixture was incubated for 1 h at 4°C with rotation. The tube was then centrifuged at 13,000 × *g* for 10 min at 4°C and the supernatant was collected as an unbound fraction. After a solution of 400 μl 10 mM Na-citrate, pH 5.5, was added to the insoluble polysaccharide pellet, the tube was centrifuged again. This washing procedure was repeated twice. The pellet was then resuspended in 200 μl of SDS-PAGE sample loading buffer and heated at 100°C for 10 min. Then, the tube was centrifuged at 13,000 × *g* for 10 min. The lysate was collected as a bound fraction. The bound and unbound fractions (10 μl, <0.25 μg protein) were visualized by SDS-PAGE and CBB R-250 staining.

## Results and Discussion

### Overall structure of ChiW-SLHd

ChiW contains 1,418 amino acids including a secretory signal peptide (Fig 1) [11]. The production of recombinant full-length ChiW protein in *E*. *coli* is challenging [11, 12]. Thus, two truncated mutant proteins have been prepared to determine three-dimensional structures, i.e., ChiW-SLHd (Val198 to Lys1418), lacking the signal peptide and SLH domains, and ChiW-CD (Val557 to Lys1418), which is composed of the two catalytic domains (Fig 1). The two monomeric proteins exhibit very similar hydrolytic activities for chitin [16]. Crystals of ChiW-CD have been obtained and preliminary X-ray crystallographic analysis of the crystals has been reported (Table 1) [16]. However, the crystal structure of ChiW-CD could not be determined solely by the MR method using the *Bacillus circulans* WL-12 chitinase A1 catalytic domain (BaChiA1CD) [24] as a reference model (amino acid identity = 47% for the 1st catalytic domain and 45% for the 2nd catalytic domain); the electron densities except for the two catalytic domains remained obscure. In this study, we have used the automated model building software (Buccaneer) [26], and the structure of ChiW-CD was completely modeled by the program and the structure was refined at 2.03 Å resolution (Table 1). On the other hand, the crystals of ChiW-SLHd were obtained in laboratories either on Earth or in space and the crystals diffracted to ~2.5 Å. The highest quality X-ray diffraction dataset was collected to 2.1 Å resolution from the crystal grown in space. The interpretable electron density map of the ChiW-SLHd structure was obtained by the MR method using the ChiW-CD structure and the Buccaneer software [26], and refined at 2.1 Å resolution (Table 1).

**Fig 2:**
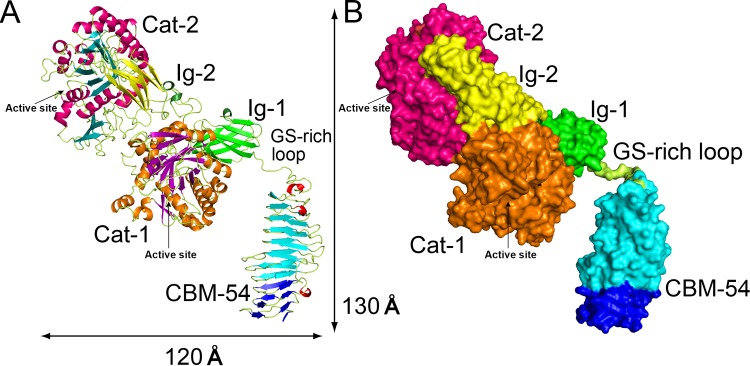
Overall structures of ChiW-SLHd. **(A** and **B**) The structures are represented as a ribbon model (**A**) and a molecular surface model (**B**). The structure is divided into three regions and six domains, a CBM-54 domain, a GS-rich loop and a catalytic region (Ig-1, Cat-1, Ig-2 and Cat-2 domains), with overall dimensions of approximately 130 × 120 × 70 Å.

Based on the crystal structure, ChiW-SLHd was functionally divided into three regions: CBM-54, the GS (glycine-serine)-rich loop and a catalytic region (Figs 1 and 2). The CBM-54 was predominantly a right-handed parallel β-helix (Ala214 to Phe449). The GS-rich loop (Gly450 to Asn463) connecting CBM-54 and Ig-1 domains consisted of consecutive glycine and serine residues with a length of 35 Å. The catalytic region is composed of four domains: 1st immunoglobulin-like fold domain (Ig-1, β-sandwich, Pro464 to Phe556), 1st GH-18 catalytic domain (Cat-1, β/α-barrel, Val557 to Lys927), 2nd Ig-like fold domain (Ig-2, β-sandwich, Ser928 to Phe1044) and 2nd GH18 catalytic domain (Cat-2, β/α-barrel, Gly1045 to Lys1418). Although the architectures in each of the six domains were classified as common protein folds, their topological and spatial arrangements for efficient chitin degradation on the cell surface are unique. The structural features of each domain are described below.

### GS-rich loop

The GS-rich loop (GGGGYGGGSGSSSN, 14 residues) connects the catalytic region and CBM-54 (Fig 2). Although similar amino acid sequences of GS-rich motifs were found in many proteins and more than 200 protein models containing the conserved motif were obtained from the PDB using the BLAST program, most structural models of the loop are missing and unavailable. In the crystal structure of ChiW, the structure of the GS-rich loop was determined. The loop is located in the catalytic cleft of Cat-1 of the symmetrically related neighbor molecule in the crystal. However, the extended structure of the loop contains no regular secondary structure features and the loop itself does not have any supportive structures. Furthermore, the GS-rich loop has a much higher average *B*-factor (93.2 Å^2^) than that of the full-length protein (33.2 Å^2^). These observations do not contradict the hypothesis that the loop is an intrinsically flexible region. ChiW is fastened on the bacterial cell surface with an SLH domain, which enables ChiW to readily collect chitin oligosaccharides into the cell. The flexible motion of the catalytic region via the GS-rich loop probably facilitates attachment of the enzyme to the molecular surface of the solid substrate chitin in an appropriate orientation.

### Structures of the GH-18 catalytic domains

**Fig 3:**
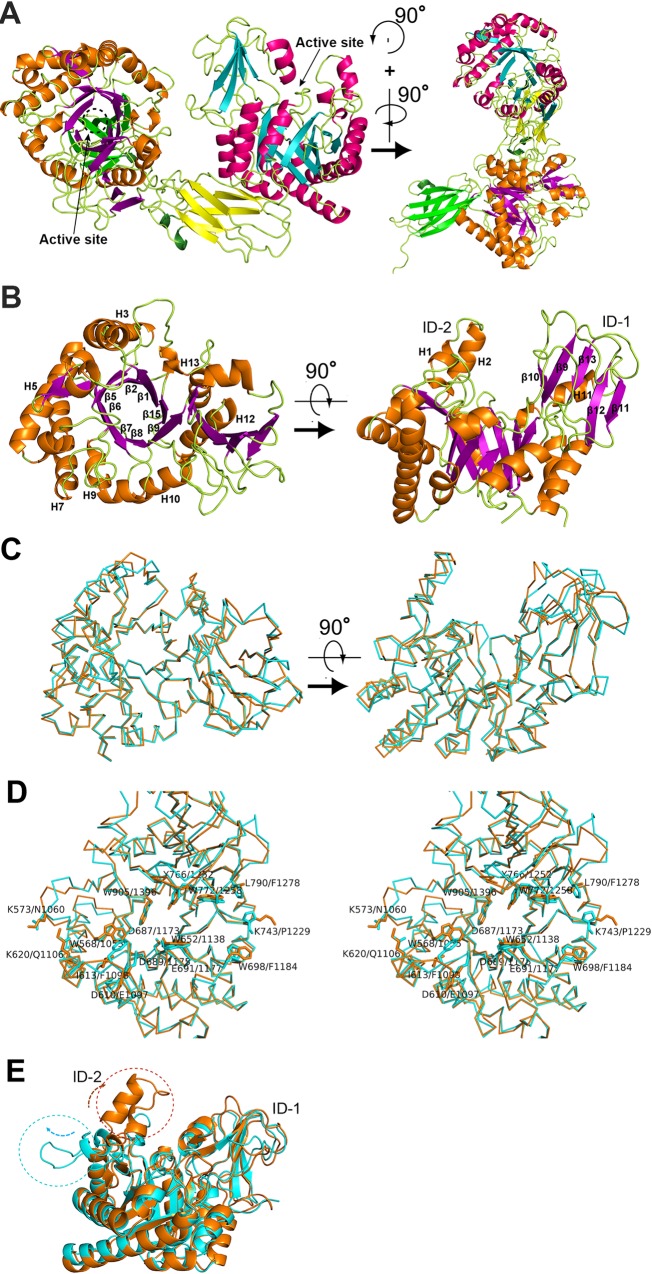
Close-up views of the structure of the catalytic region of ChiW. **(A**) The overall catalytic region is shown as a ribbon model; front view (left figure) and side view (right figure). (**B**) Left figure, top view of the ChiW Cat-1 domain. The secondary structure elements (α-helices and β-strands) of the β_8_/α_7_-barrel are indicated by numbers. Right figure, side view of the ChiW Cat-1 domain. The secondary structure elements (α-helices and β-strands) of the extra subdomains (ID-1 and ID-2) are indicated by numbers. (**C**) Structural comparison of Cat-1 (orange) and Cat-2 (cyan). The three-dimensional structures are very similar (rmsd = 1.0 Å) with high amino acid sequence similarity (56% identity). (**D**) A comparison of the catalytic cleft of ChiW Cat-1 (orange ribbon model), and Cat-2 (cyan ribbon model). The amino acid residues (stick models), Trp568/Trp1055, Trp652/Trp1138, Asp687/Asp1173, Asp689/Asp1175, Glu691/Glu1177, Tyr766/Tyr1252, Trp772/Trp1258, Trp905/Trp1396, are near identical in the two active clefts. The amino acid residues (stick models), Lys573/Asn1060, Asp610/Glu1097, Ile613/Phe1098, Lys620/Gln1106, Trp698/Phe1184, Lys743/Pro1229 and Leu790/Phe1278, are located at the edge of the clefts. (**E**) Structural comparison of ChiW (orange) and BaChiA1CD (PDB ID: 1ITX; cyan). Their β_8_/α_7_-barrel core structures and ID-1 are similar, but significant differences exist in ID-2. The ChiW ID-2 forms the wall of the cleft, whereas the identical area of BaChiA1CD is positioned outside of the cleft.

In the catalytic region (Fig 3A), the three-dimensional structure of Cat-1 (Fig 3B) closely resembled that of Cat-2, consistent with their high degree of amino acid sequence similarity (56% identity). Superimposition of the 355 residues of Cat-1 and Cat-2 gave a root-mean-square deviation (rmsd) of 1.0 Å (Fig 3C) and the surface models of the two clefts are similar (S1 Fig). Cat-1 possesses 13 β-strands and 13 α-helices and forms a β_8_/α_7_-barrel as a core structure (Fig 3B), which is commonly observed in the GH-18 family as a canonical triosephosphate isomerase fold [36]. Besides the core β_8_/α_7_-barrel, the ChiW catalytic domain has an additional two subdomains, an (α+β)-insertion domain and insertion domain-2 (Fig 3B). The insertion domains are characteristic subdomains for chitinases that form the walls of the substrate binding clefts or tunnels [37, 38]. The (α+β)-insertion domains are commonly found in many chitinases. The (α+β)-insertion domain (ID-1; around Leu768–His779 and Phe815–Ala885 of Cat-1) of ChiW is composed of an anti-parallel β-sheet and α-helix. The insertion domain-2 (ID-2; around Gly591–Pro625 of Cat-1) consists of a long loop and two helices (Fig 3B). The two subdomains protrude from the core β/α-barrel and form the walls of a deep active cleft of approximately 42 Å in length and 26 Å in depth (Fig 3B). No major differences exist between the overall structures of the two catalytic domains (Fig 3C) and the amino acid residues at the center of the two active sites, i.e., Trp568/Trp1055, Trp652/Trp1138, Asp687/Asp1173, Asp689/Asp1175, Glu691/Glu1177, Tyr766/Tyr1252, Trp772/Trp1258, Trp905/Trp1396, are almost identical (Fig 3D). However, some amino acid residues at the edge of the clefts, such as Lys573/Asn1060, Asp610/Glu1097, Ile613/Phe1098, Lys620/Gln1106, Trp698/Phe1184, Lys743/Pro1229 and Leu790/Phe1278, are not identical (Fig 3D). These residues make small differences to the shape of the clefts and may influence enzymatic properties. Further studies are needed to evaluate the catalytic roles of Cat-1 and Cat-2 with respect to these structural differences.

Based on the structural similarity observed with the DALI program, BaChiA1CD exhibited the highest degree of similarity to the ChiW catalytic domains (Fig 3E). The rmsd was 1.3 Å (or 1.4 Å) for superimpositioning 333 (or 335) residues of Cat-1 (or Cat-2) onto those of BaChiA1CD with relatively high amino acid sequence similarity (identity = 47% for Cat-1, 45% for Cat-2). The core β/α-barrel and ID-1 structures are similar between BaChiA1CD and ChiW (Fig 3E). However, ChiW ID-2 forms a high wall along the active site cleft. In the corresponding region of BaChiA1CD, instead of ID-2, long loops locate outside of the catalytic domain (Fig 3E). The cleft architecture for substrate binding is described below.

### Structures of the Ig-like fold domains

**Fig 4:**
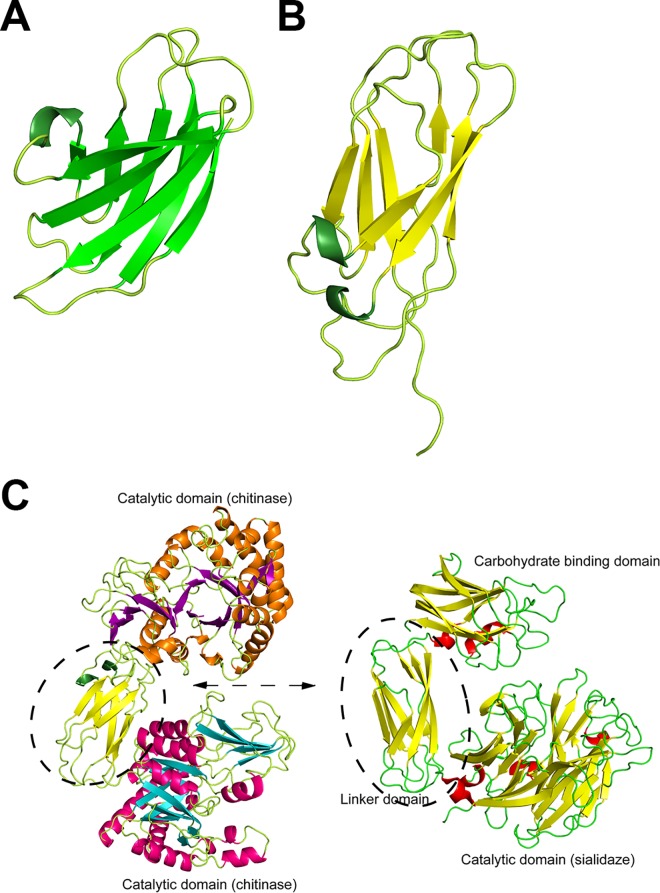
Ig-like fold domains of ChiW. (**A** and **B**) The Ig-1 (**A**), and Ig-2 (**B**) domains are shown as ribbon models. (**C**) Structural comparison of ChiW-CD (left figure) and *Micromonospora viridifaciens* sialidase (PDB ID: 2BQ9) (right figure). The Ig-2 domain and the bacterial sialidase linker domain are very similar structures (rmsd, 2.1 Å).

There are two Ig-like fold domains in addition to the GH-18 catalytic domains in the catalytic region (Fig 3A). Although the architectures of Ig-1 and Ig-2 are classified as Ig-like folds [39, 40], there is little or no similarity in their amino acid sequences. The Ig-1 structure is composed of an eight-stranded β-sandwich fold containing two four-stranded antiparallel β-sheets closely stacked upon each other (Fig 4A). The structure of Ig-2 possesses a seven-stranded β-sandwich with two antiparallel β-sheets composed of three and four β-strands (Fig 4B). Their amino acid sequences also had no significant similarities to other known proteins or domains. However, structurally similar proteins to Ig-1 in the PDB were found; besides the expected immunoglobulin light chains, a number of animal adhesion domains of transmembrane receptor proteins [41] were identified. Adhesion domains interact with other proteins in cell–cell adhesion processes. In the case of Ig-2, some linker domains of enzymes were identified as structurally similar proteins. In particular, a bacterial sialidase linker domain [42] showed the highest similarity to Ig-2. Superposition of the whole sequence of Ig-2 and the bacterial sialidase linker domain (PDB ID: 2BQ9) gave an rmsd of 2.1 Å (Fig 4C), despite the overall lack of amino acid sequence similarity (< 10%). The bacterial sialidase linker domain connects the carbohydrate binding and catalytic domains [42].

### Active cleft and chitin degradation manner of ChiW

**Fig 5:**
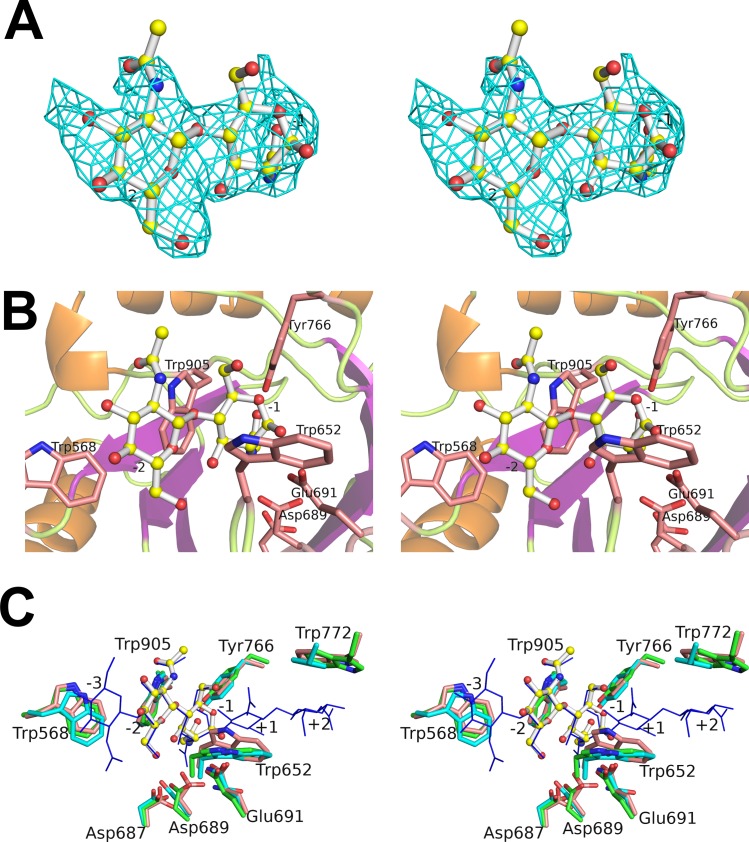
Active site of ChiW. (**A** and **B**) The reaction product is located in the binding cleft of ChiW Cat-1. Electron density of the reaction product (GlcNAc)_2_ (stick model: carbon atoms, yellow; oxygen atoms, red; and nitrogen atoms, blue) in the omit (*F*o–*F*c) map (cyan) (**A**) was calculated without the substrate and contoured at the 3.0-σ level. The ChiW residues (**B**) that interact with the product are represented by pink stick models (oxygen atoms, red and nitrogen atoms, blue). The numbers (−2 and −1) indicate the subsite positions. Other numbers indicate the amino acid residues. (**C**) The comparison of the catalytic cleft residues at the 5 subsites (−3 to +2) of ChiW Cat-1 (pink), Cat-2 (green) and SerChiA (cyan). The numbers indicate the amino acid residues of ChiW Cat-1.

To determine the implications of the active cleft of ChiW, we attempted, but failed, to prepare crystals of ChiW-SLHd bound to substrates or products by soaking or cocrystallization. This was also the case when using the inactive ChiW mutant that has substitutions of Gln for Glu (i.e., the E691Q and E1177Q double mutant). However, the crystal of ChiW-CD in complex with the reaction product (GlcNAc)_2_ was obtained through cocrystallization with the substrate (GlcNAc)_3_ (Fig 5A and 5B, Table 1). The production of (GlcNAc)_2_ from (GlcNAc)_3_ with ChiW-CD was confirmed by HPLC analysis (S2 Fig). Although crystals of ChiW-CD-DM (the E691Q and E1177Q double mutant) were also obtained in the presence of (GlcNAc)_4_, (GlcNAc)_5_ or (GlcNAc)_6_, the electron density maps corresponding to the substrates were too weak and complicated to interpret because of their diversity and the heterogeneity in substrate binding modes within the active site. The structure of the ChiW-CD-product complex contained one (GlcNAc)_2_ molecule at the bottom of the deep cleft of Cat-1, indicating that it occupied two subsites, −1 and −2 (Fig 5B). In an |*F*_o_|–|*F*_c_| electron density map of the product complex, another peak was found around the subsites +1 to +3, although the electron densities of the peak were too weak to construct precise structure models. The subsites are specified in accordance with the nomenclature described by Davies *et al*. [43]. The puckering parameters [44] of the bound (GlcNAc)_2_ were *Q* = 0.59 Å, *Θ* = 65° and *Φ* = 259° for GlcNAc at the −1 subsite, and *Q* = 0.60 Å, *Θ* = 14° and *Φ* = 61° for GlcNAc at the −2 subsite. Therefore, the −1 subsite GlcNAc adopts a screw-boat conformation (^1^*S*_5_) with the β-anomer and the −2 subsite GlcNAc adopts a stable chair conformation (^4^*C*_1_). The ring distortion at the −1 subsite has been observed in the complex of other glycosidases [45] and is critical in the GH-18 chitinase reaction mechanism [36]. In an attempt to further elucidate the substrate recognition by ChiW, a chitin oligosaccharide was superimposed onto the active cleft of Cat-1 (Fig 5C) based on the *Serratia marcescens* E315Q mutant chitinase A (SmChiA) structure in complex with octa-*N*-acetylchitooctaose (GlcNAc)_8_ [46] and conserved amino acid residues of the chitinases. The rmsd was 1.2 Å for superposition of the catalytic domains of ChiW and SmChiA, even though the sequences show moderate sequence identity (29%). The stable conformations of the −2 subsite GlcNAc residues of the two structures superimpose well, and the conformations of the distorted sugar rings of GlcNAc residues at the −1 subsite of the two complex structures are almost the same (Fig 5C). However, a significant difference is observed in the orientations of their *N*-acetyl groups of the distorted residue. In contrast to the *N*-acetyl group of ChiW forming a hydrogen bond to Tyr766 (2.4 Å) and the O atom of the *N*-acetyl group being adjacent to the C1 atom and within a hydrogen bond distance (2.9 Å), those of SmChiA face an opposite orientation and form a hydrogen bond to Gln315 (2.4 Å). The difference may result from structure determination of an inactive mutant (E315Q) of SmChiA. The superimposed structures also indicated the important residues for ChiW substrate binding at the 5 subsites (−3 to +2) (Fig 5C). The SmChiA residues important for saccharide binding, Trp167 at the −3 subsite, Trp539 at the −1 subsite, Trp275 at the +1 subsite and Phe396 at the +2 subsite [38, 46] corresponded to the ChiW residues Trp568/Trp1055, Trp905/Trp1396, Trp652/Trp1138 and Trp772/Trp1258 for Cat-1/Cat-2, respectively. The SmChiA catalytic residues Tyr390, Asp311, Asp313 and Glu315 corresponded to Tyr766/Tyr1252, Asp687/Asp1173, Asp689/Asp1175 and Glu691/Glu1177 for Cat-1/Cat-2, respectively. These conserved resemblances indicate that ChiW possesses a catalytic mechanism that is similar to SmChiA and general GH-18 chitinases. Based on the generally accepted mechanism, chitin hydrolysis by ChiW is likely to be assisted by the *N*-acetyl group of the substrate as a nucleophile and the glutamate residues, E691 for Cat-1 and E1177 for Cat-2, which function as a general acid [36]. The side chain of Asp689 forms a hydrogen bond with the general acid, Glu691 (2.7 Å) in the ChiW complex structure (ChiW-CD/(GlcNAc)_2_). In contrast, in the apo structure (ChiW-CD), the side chain of Asp689 orients to form a hydrogen bond with Asp687 (2.4 Å). The proton donation from Asp689 to Glu691 is a common structural feature in bacterial chitinases [36, 47]. This catalytic mechanism is also supported by the result from the double mutant enzyme of the catalytic residues, ChiW E691Q/E1177Q, which has no efficacious activity, as described before [12].

**Fig 6:**
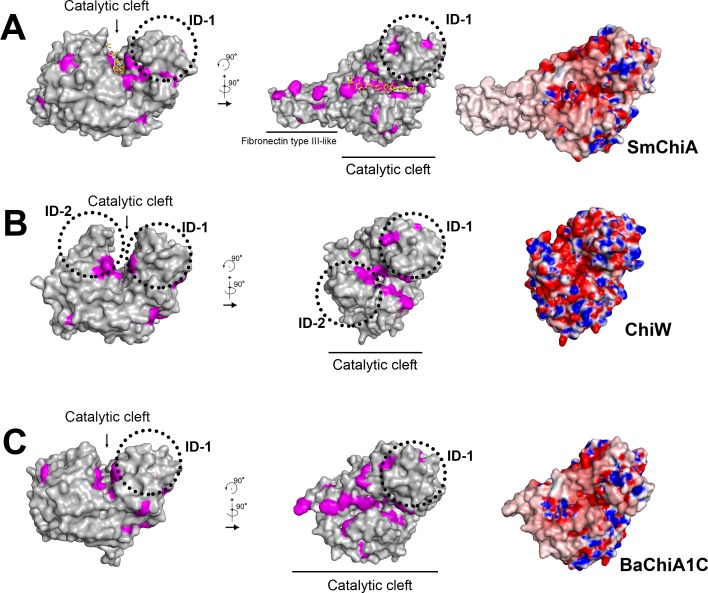
Surface structure of ChiW catalytic domain. (**A-C**) The surface models of SmChiA (**A**), Cat-1 of ChiW (**B**) and BaChiA1CD (**C**). The side chains of the aromatic residues (Trp, Phe and Tyr) are shown in magenta. Electrostatic potentials at pH 7 are also represented. The +8 to –8 kT/e potential isocontours are shown as blue to red surfaces, respectively. ChiW catalytic domains have characteristic subdomains (ID-1 and ID-2) that form deep and short clefts surrounded by negative charges.

On the other hand, many glycoside hydrolases, in particular polysaccharide-degrading enzymes, have one or more carbohydrate-binding modules in addition to catalytic domains. In the GH-18 family chitinases, chitin-binding modules often locate along their catalytic domains and assist in the processive degradation of one chitin chain [6, 7, 37, 38, 46]. For example, SmChiA has one fibronectin type III-like domain as a chitin-binding module that makes a minus subsite (Fig 6A), which leads to enzyme degradation of chitin from the reducing ends with the production of (GlcNAc)_2_ residues, whereas *Serratia marcescens* ChiB (SmChiB), with a chitin-binding module on the opposite side for a plus subsite, degrades the polymer from the nonreducing ends and also produces (GlcNAc)_2_ residues. However, ChiW catalytic domains, Cat-1 and Cat-2, had no such fibronectin type III-like domain or chitin-binding module (Fig 6B). In the solved structure, the two clefts cross each other at approximately right angles (Fig 3A). Aromatic residues are located on the surface of Ig-1, i.e., Tyr486, Tyr537 and Phe 556, and Tyr939, Tyr948, Tyr1000 and Phe1044 on the surface of Ig-2 (S3 Fig), and the Ig-1 and Ig-2 domains might be functional substitutions of the chitin-binding module. However, they are too distal from the catalytic clefts to function as a chitin-binding module (Fig 3A and S3 Fig). The Ig-1 and Ig-2 domains might serve as linkers or scaffolds for the two catalytic domains. Ig-1 interacts with the back face of the substrate-binding cleft of Cat-1 via loop-loop interactions (Fig 3A). The loops on one side of Ig-2, the ID-1 and two α-helices of Cat-1 participate in the interface between Cat-1 and Ig-2, while the β-sheet side of Ig-2 contacts the opposite side of the substrate-binding cleft of Cat-2 (Fig 3A). These associations, dominated by α-helices and β-sheets, also occur in cohesin (Ig fold, β-sheets)-dockerin (α-helices) interactions of the cellulosome involved in the organization of individual enzymatic subunits into a multi-enzyme assembly [48]. The substrate binding sites of ChiW are surrounded by aromatic residues for chitin binding, which is similar to other chitinases, but are different in length to those of SmChiA composed of catalytic and fibronectin type III-like domains; the binding sites of ChiW are shorter in length and no carbohydrate-binding surface is found in the neighborhood (Figs 3B and 6A–6C). Furthermore, the walls of active clefts of ChiW are more negative than those of other chitinases (Fig 6), which may define the substrate recognition properties or degradation mechanism of ChiW.

In a previous study, the specific activity of ChiW-SLHd against colloidal chitin was 4.9 U mg^‒1^, as determined by the quantification of the reducing ends (aldehyde groups newly produced by the reaction) with 3-methyl-2-benzothiazolinone hydrazone [12]. In this study, we measured (GlcNAc)_2_ residues, the repeating unit of chitin, released from the end of the chitin chain in the reaction with colloidal chitin using liquid chromatography, and it was quantified as 2.1 U mg^‒1^. The difference in the two values may indicate that the two catalytic domains of ChiW that resemble each other work as an endolytic enzyme with low processivity. In examining the values, approximately five reducing ends and two (GlcNAc)_2_ residues are produced, and the number of sequential catalytic cycles of ChiW without dissociation from a single chain was one to three per chitin chain. In other words, ChiW releases one or two (GlcNAc)_2_ residues from one chain with a processive action. Although the active site residues of ChiW are quite similar to those of SmChiA producing (GlcNAc)_2_ from the reducing ends as an exo-type chitinase, as described above, ChiW shows low processive movements. The lack of a general chitin-binding module and the short active clefts (Fig 3B) probably enable ChiW to transfer from chain to chain with low processivity.

### Structure of CBM-54

**Fig 7:**
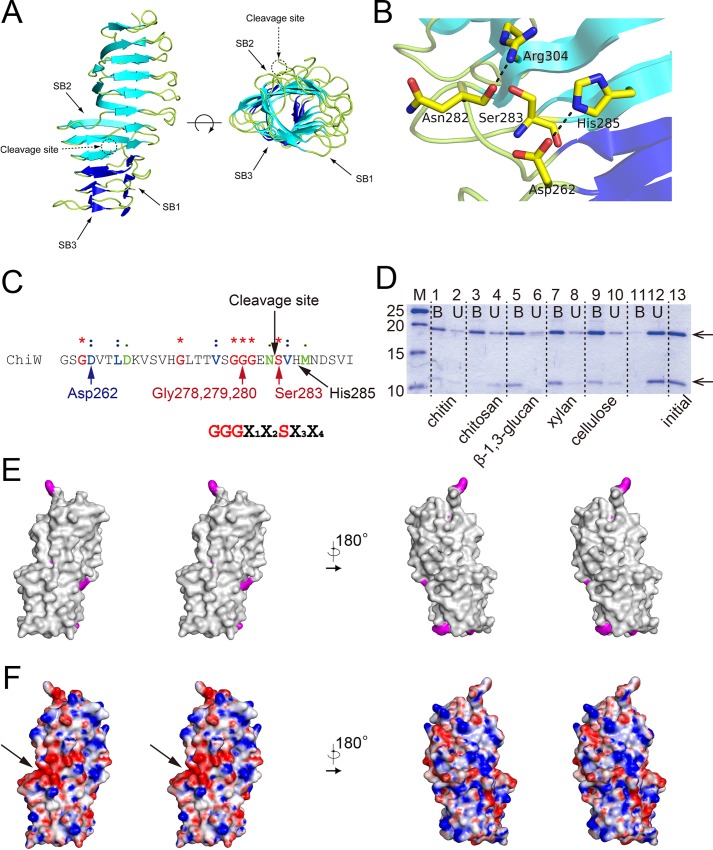
Properties of the CBM-54 of ChiW. (**A**) The CBM-54 structure is shown as a ribbon model, front view (left figure) and top view (right figure). The right-handed β-helix structure consists of three distorted faces (SB1, SB2 and SB3). The cleavage site is located at one-third of the total distance from the bottom and between the blue and cyan ribbon models. (**B**) A close-up structure of the limited proteolysis site. The nearest residues of the cleavage site are shown as stick models (carbon atoms, yellow; oxygen atoms, red; and nitrogen atoms, blue), which form hydrogen bond networks. (**C**) The amino acid sequence of the cleavage site. Identical amino acid residues among the conserved proteins are indicated by asterisks, whereas colon and period characters indicate highly conserved residues. The sequence motif can be recognized as G-G-G-X1-X2-S-X3-X4 (the cleavage site is between X2 and S; X1: anything; X2: N, Q, D, or H; X3: V or I; X4: H, K, L, N, Y, or V). (**D**) SDS-PAGE analysis of the bound CBM-54 of ChiW (10 μg) with insoluble polysaccharides (2 mg). Protein bands were stained with CBB R-250. Lane M, molecular mass standards (25, 20, 15 and 10 kDa); lanes 1 and 2, bound or unbound CBM-54 with chitin; lanes 3 and 4, with chitosan; lanes 5 and 6, with β-1,3-glucan; lanes 7 and 8, with xylan; lanes 9 and 10, with cellulose; lanes 11 and 12 with no polysaccharide; lane 13, the purified CBM-54 domain (0.25 μg). The upper arrow corresponds to a CBM-54 domain fragment of ~18 kDa and the lower arrow to a ~9 kDa fragment. (**E**, **F**) The surface models of CBM-54 ChiW. The side chains of the aromatic residues (Trp, Phe and Tyr) are shown in magenta. The molecular surface has no distinctive cleft or patch surrounded by aromatic residues. Electrostatic potentials at pH 7 are also presented (**F**). The +12 to –12 kT/e potential isocontours are shown as blue to red surfaces, respectively. The negatively charged patch exists on the central part of the β-helix structure and is indicated by a black arrow.

We report here the first three-dimensional structure of CBM-54. CBM-54 family proteins, containing 79 bacterial proteins, are established in the CAZy database as a cell-wall-carbohydrate binding module. The domain folds into a 12 coiled and right-handed β-helix structure (Fig 7A). There are 34 β-strands that form three parallel β-sheets, named SB1 (12 β-strands), SB2 (12 β-strands) and SB3 (10 β-strands), making three distorted faces (Fig 7A). The electron density maps corresponding to the N-terminal 39 residues (additional peptide from the expression vector and Val198 to Ile213) were faintly observed along the SB1 face and in the cleft of the neighbor Cat-2 molecule. Currently, the function of CBM-54 from *Ruminiclostridium thermocellum* (formerly known as *Clostridium thermocellum****)*** DSM 1237 lichenase A (Lic16A) [49, 50] and *Paenibacillus* sp. CCRC 17245 endo-β-1,3-glucanase (LamA) [51] have been reported to bind specifically to cell wall carbohydrates, such as chitin, chitosan, xylan and glucan. However, the right-handed parallel β-helix fold is found in some enzymes that react with polysaccharides, such as pectate lyase C [52] and the surface-exposed domains of cell surface proteins. Indeed, putative pectinase of *Parabacteroides distasonis* (rmsd of 2.2 Å and 8% sequence identity; PDB ID: 3LYC), *Enterobacteria* phase P22 tail spike protein (rmsd of 2.3 Å and 16% sequence identity; PDB ID: 1QQ1) [53] and *Bordetella pertussis* P.69 pertactin (rmsd of 2.1 Å and 10% sequence identity; PDB ID: 1DAB) [54] were identified as structural homologs to CBM-54 in the PDB.

ChiW, which has been suggested to be a monomer enzyme by gel permeation chromatography, is cleaved between Asn282 and Ser283 at CBM-54, as described before [12]. The native ChiW is localized in the cell fraction of *P*. str. FPU-7; as judged by western blotting analysis [11]. The cleavage proceeds through the purification of recombinant or native ChiW proteins. The trigger for self-splicing remains unresolved. The CBM-54 of Lic16A also undergoes specific cleavage between Asp and Ser, and the two truncated polypeptide chains also exist as a monomeric enzyme [49]. In the crystal structure, the location of this cleavage site is on the SB2 face and in front of the 11th β-strand at the fourth coil from the N-terminus (Fig 7A and 7B). Although it is unclear whether the two cleaved segments of ChiW (120 kDa and 30 kDa) coexist on the cell surface, the crystal structure also indicated that the two polypeptides bound tightly to each other with 13 hydrogen bonds between the third and fourth coils and they retain the β-helix fold. Based on careful examination of the cleavage site, amino acid residues Ser283, His285, Asp262 and Arg304 are located in the region (Fig 7B). Successive glycine residues near the cleavage site presumably confer conformational flexibility to this site (Fig 7C). This limited proteolysis could explain self-splicing with the hydroxyl group of Ser283 as a nucleophile [55]. The amino acid residues of this cleavage site, Asn-Ser, have been found in various self-splicing proteins, supporting this inference. Although detailed analysis is necessary to confirm whether this process results from self-splicing or other specific proteases, it is generally accepted that the side chain of Ser is engaged as a nucleophile in self-cleaving proteins, such as inteins [55] and hedgehog proteins [56]. The *Clostridium difficile* cell wall protein CwpV also undergoes self-cleavage via the hydroxyl group of threonine [57]. Although the catalytic residue of CwpV is not serine, but threonine, and there is no sequence similarity between CwpV and ChiW, both proteins are expressed on the cell surfaces of Gram-positive bacteria. In addition, the predicted secondary structure of CwpV shows that the cleavage site is positioned on the edge of a β-strand [57], as observed for ChiW.

The amino acid residues near the processing site of the CBM-54 domain (~30 residues) are highly conserved in a large number of proteins (including predicted proteins) of Gram-positive bacteria such as *Paenibacillus*, *Caldicellulosiruptor*, *Bacillus*, *Clostridium*, *Ruminiclostridium*, *Desulfosporosinus*, *Thermoanaerobacterium*, *Tepidanaerobacter* and *Ruminococcaceae* species (S4 Fig). Most of these conserved residues are involved in the stability of the β-helix because their side chains face the center of the coil. Among the residues in proximity to the cleavage site, Ser283 and three Gly residues (Gly278, Gly279 and Gly280) are essentially conserved, whereas Asp262, Asn282 and His285 are highly conserved with Glu or Asn at Asp262, Gln, His, or Asp at Asn282, and Lys, Leu, Asn, Tyr, or Val at His285. The detailed sequence motif is G-G-G-X_1_-X_2_-S-X_3_-X_4_ in this region (the cleavage site is between X_2_ and Ser; X_1_: anything; X_2_: N, Q, D, or H; X_3_: V or I; X_4_: H, K, L, N, Y, or V) (Fig 7C).

### Insoluble polysaccharide binding capability of the CBM-54 of ChiW

The protein fold of CBM-54 is not similar to known CBM structures but to those of extracellular enzymes, as described above. The polysaccharide degradation assays were carried out with a recombinant protein composed of the CBM-54 domain (Val198 to Phe449, Fig 1). However, the domain had no detectable activity towards the polysaccharide components of cell walls, such as chitin, chitosan, cellulose, xylan and β-1,3-glucan. Then, to determine whether the CBM-54 of ChiW was capable of binding to insoluble polysaccharides, as observed for other CBM-54 domains [49, 51], pull-down assays were performed with 10 μg CBM-54 of ChiW against 2 mg chitin or other non-substrate insoluble polysaccharides, chitosan, β-1,3-glucan, xylan and cellulose. The CBM-54 of ChiW bound to these polysaccharides (Fig 7D), which is in agreement with other characterized CBM-54 proteins such as Lic16A and LamA.

Although the CBM-54 of ChiW binds insoluble polysaccharides, its molecular surface has no distinct cleft or patch surrounded by aromatic residues that would function as a potential polysaccharide-binding site (Fig 7E). Instead of aromatic residues, a negatively charged patch exists on a shallow cleft-like region and is located in the central part of the β-helix structure (Fig 7F).

### Conservation of the CBM-54 of ChiW in soil-dwelling Gram-positive bacteria

The limited proteolysis motif (~30 residues) of CBM-54 is highly conserved among a large number of proteins, as described above. Among the characterized CBM-54, the sequence motif is also highly conserved (ChiW and Lic16A, 48%; ChiW and LamA, 45%; Lic16A and LamA, 55% identity). However, full-length sequence similarities of the characterized CBM-54 are very low (ChiW and Lic16A, 27%; ChiW and LamA, 18%; Lic16A and LamA, 23% identity) (S5 Fig). A phylogenetic tree was constructed using the amino acid sequences of CBM-54 domains listed in the CAZy database (S6 Fig). In the tree, the location of CBM-54 of ChiW is very far from those of CBM-54 of Lic16A or LamA. The absence of sequence similarities suggests differences in their functional properties. On the other hand, a number of amino acid sequences similar to CBM-54 of ChiW are found in the protein sequences of Gram-positive soil-dwelling bacteria, e.g., *Paenibacillus*, *Desulfotomaculum*, *Bacillus*, *Caldicellulosiruptor*, *Tepidanaerobacter*, *Acetobacterium*, *Clostridium*, *Caldicellulosiruptor* and *Ruminococcaceae* species (S1 Table). Many of these proteins are multi-modular and are classified as fungal- or plant-cell wall polysaccharide-degrading enzymes, e.g., GH-18 chitinase, GH-16 β-glucanase, GH-26 mannosidase and GH-43 β-xylosidase, with SLH domains present on cell surfaces. The CBM-54 domains were predicted to be located between the SLH domain and polysaccharide (glucan, mannan, or xylan)-hydrolyzing domain (S1 Table). This multi-modular protein architecture indicates that polysaccharide degrading enzymes with SLH and CBM-54 domains are common devices used by Gram-positive bacteria to degrade cell walls efficiently.

## Conclusions

ChiW is induced by feeding chitin or (GlcNAc)_2_ to the FPU-7 strain of *Paenibacillus* sp. and is presented on the bacterial peptidoglycan layer with SLH domains [11]. In the catalytic region, ChiW has two GH-18 chitinase domains with similar amino acid sequences (56% identity) and the crystal structure of the domains indicate that they are almost identical (rmsd = 1.0 Å) (Fig 3C and S1 Fig). The presence of two catalytic domains in a single ChiW protein appears to be the result of a gene duplication event. Unfortunately, the individual catalytic domains could not be prepared as stable enzymes. The functional differences of the two catalytic domains have not been clearly characterized. The two Ig-like fold domains, Ig-1 and Ig-2, bind to the catalytic domains and may function to stabilize these domains. The reason why the enzyme has multiple catalytic domains remains unclear. In the gene of other *Paenibacillus* sp., one protein (*Paenibacillus* sp. HGF7, ZP_08511493) is predicted to have three chitinase catalytic domains with SLH and CBM-54 domains (S1 Table). *P*. str. FPU-7 is a rod-shaped bacterium (length, 1–10 μm and diameter, 0.25–1.0 μm). Stacking enzymes to the cell exterior increases the number of enzymes proximate to the cell surface. Since the surface area of the cell is limited, this stacking of enzymes near the cell surface appears to be a good strategy for bacteria. The highly flexible GS-rich loop endows the catalytic region with flexibility to anchor to the target chitin polysaccharides present in fungal cell walls (Fig 2). The cylindrical CBM-54 domain interacts with some cell wall polysaccharides (Fig 7A and 7D). The structure of cell walls consists of various polysaccharides is therefore a complex network of these polysaccharides. Some cellulose binding modules can attach to noncellulase catalytic domains, e.g., xylanase, mannase, or pectinase [58]. When carbohydrate binding modules recognize polysaccharides, whether substrate or non-substrate, the proximity effect enhances the efficiency of the catalytic domains. Once the catalytic regions catch the target chitin polysaccharides present in fungal cell walls, the CBM-54 domain can be rigidly attached to the coexisting cell wall polysaccharides, which enables the bacteria to dock with the target cell wall. However, this continuous harboring action on the surface would be inefficient for the bacteria to degrade the target cell wall or inner contents owing to limitations with respect to movement. The cleavage site of the CBM-54 domain would help bacteria detach from the cell wall or move to other areas. We envisage that the catalytic domain Cat-1 breaks down one chitin chain while Cat-2 positioned axially to Cat-1 to attack continuously surrounding chains with low processive activity. Our sequence homology analysis (S1 Table) and characterization of the other CBM-54 domains [49, 51] indicate that a number of gram-positive soil-dwelling bacteria possess similar cell-surface-expressed multi-modular enzymes for cell wall polysaccharide degradation.

## Supporting Information

S1 Fig Molecular surface models of catalytic domains, Cat-1 and Cat-2. The side chains of the aromatic residues (Trp, Phe and Tyr) are shown in magenta. The shapes of the clefts are similar.
(PDF)

S2 Fig HPLC profiles for ChiW-CD catalyzed hydrolysis of (GlcNAc)_3_. The reaction solution consisted of 5 mM sodium acetate buffer (pH 5.5), 2 mM (GlcNAc)_3_ and 100 nM ChiW-CD in a 100 μl reaction volume. The reaction was terminated by withdrawing 10 μl aliquots from the reaction solution and then adding 10 μl acetonitrile at 1 (black line), 3 (green line), 10 (blue line) and 20 min (red line). The mixture (5 μl) was subsequently separated on a TSKgel Amide-80 column (4.6 × 250 mm; Tosoh Co., Tokyo, Japan) using 70% (v/v) acetonitrile and detected at 210 nm. The alpha and beta anomers of GlcNAc, (GlcNAc)_2_ and (GlcNAc)_3_ were separated by this column under these conditions.
(TIF)

S3 Fig **Molecular surface models of Ig-1 and Cat-1 (A) and Cat-1, Ig-2 and Cat-2 (B).** Ig-1 and Ig-2 domains are located on the opposite side of the catalytic clefts. The aromatic residues located on the surface of Ig-1 (Tyr486, Tyr537 and Phe556) and surface of Ig-2 (Tyr939, Tyr948, Tyr1000 and Phe1044) are shown in magenta.
(PDF)

S4 Fig Amino acid sequences near the cleavage site of ChiW and corresponding residues of other proteins. The numbers in the left-hand column are the protein accession numbers (ZP_07902840: S-layer domain protein [*Paenibacillus vortex* V453]; YP_004027356: s-layer domain-containing protein [*Caldicellulosiruptor kristjanssonii* I77R1B]; YP_004797824: S-layer protein [*Caldicellulosiruptor lactoaceticus* 6A]; ZP_07387876: S-layer domain protein [*Paenibacillus curdlanolyticus* YK9]; YP_003008964: S-layer protein [*Paenibacillus* sp. JDR-2]; ZP_08511493: hypothetical protein HMPREF9413_5209 [*Paenibacillus* sp. HGF7]; EIJ83768: S-layer domain protein [*Bacillus methanolicus* MGA3]; ZP_09079447: S-layer domain-containing protein [*Paenibacillus elgii* B69]; YP_003009590: glycoside hydrolase family 16 [*Paenibacillus* sp. JDR-2]; ABJ15796: endo-beta-1,3-glucanase [*Paenibacillus* sp. CCRC 17245]; YP_005047467: beta-propeller domain-containing protein, methanol dehydrogenase [*Clostridium clariflavum* DSM 19732]; ZP_08278755: hypothetical protein HMPREF9412_0339 [*Paenibacillus* sp. HGF5]; YP_003240334: S-layer domain-containing protein [*Paenibacillus* sp. Y412MC10]; YP_006468477: cell wall-binding protein [*Desulfosporosinus acidiphilus* SJ4]; YP_006189237: mannan endo-1,4-beta-mannosidase [*Paenibacillus mucilaginosus* K02]; YP_004640549: mannan endo-1,4-beta-mannosidase [*Paenibacillus mucilaginosus* KNP414]; YP_005312442: mannan endo-1,4-beta-mannosidase [*Paenibacillus mucilaginosus* 3016]; YP_002506677: S-layer protein [*Clostridium cellulolyticum* H10]; YP_003850806: Mannan endo-1,4-beta-mannosidase [*Thermoanaerobacterium thermosaccharolyticum* DSM 571]; YP_004310855: glucan endo-1,3-beta-D-glucosidase [*Clostridium lentocellum* DSM 5427]; YP_006391314: glycoside hydrolase family 26 [*Thermoanaerobacterium saccharolyticum* JW/SL-YS485]; YP_004459776: S-layer domain-containing protein [*Tepidanaerobacter acetatoxydans* Re1]; ZP_08191088: S-layer domain-containing protein [*Clostridium papyrosolvens* DSM 2782]; ZP_09079445: hypothetical protein PelgB_33676 [*Paenibacillus elgii* B69]; ZP_08420347: putative S-layer homology domain protein [*Ruminococcaceae bacterium* D16]; EIC10715: glycoside hydrolase family 16, partial [*Ruminiclostridium thermocellum* AD2]; ZP_06250297: glycoside hydrolase family 16 [*Ruminiclostridium thermocellum* JW20]; CAC27412: endo-1,3(4)-beta-glucanase [*Ruminiclostridium thermocellum* YP_001039201: glycoside hydrolase family 16 [*Ruminiclostridium thermocellum* ATCC 27405]; ZP_05428042: glycoside hydrolase family 16 [*Ruminiclostridium thermocellum* DSM 2360]). Identical amino acid residues among the conserved proteins are indicated by red asterisks. The blue colons and green periods indicate conserved residues.
(PDF)

S5 Fig Amino acid sequence alignment of the CBM-54 of ChiW and other functionally characterized CBM-54 domains using ClustalW. ChiW CBM-54, CBM-54 domain of *Paenibacillus* sp. str. FPU-7 ChiW; Lic16A CBM-54, CBM-54 domain of *Ruminiclostridium thermocellum* DSM 1237 lichenase A; and LamA CBM-54, CBM-54 domain of *Paenibacillus* sp. CCRC 17245 endo-β-1,3-glucanase (LamA). Identical amino acid residues among the conserved proteins are indicated by asterisks, whereas colon and period characters indicate conserved residues. The limited proteolysis motif is indicated by a red box. The red and blue characters indicate highly conserved residues. The amino acid residues, Asp262, Ser283, His285 and Arg304 are located at the cleavage site of ChiW.
(PDF)

S6 Fig Phylogenetic tree based on amino acid sequence alignment for CBM-54 domains. The amino acid sequences of 74 CBM-54 family members were taken from the CAZy database and aligned by ClustalW. The tree is constructed by the neighbor-joining method with 1,000 bootstrap replications. Numbers at branching points refer to bootstrap values. The characterized CBM-54 domains, *Paenibacillus* ChiW CBM-54 domain, *Paenibacillus* sp. CCRC 17245 LamA CBM-54 domain, Lic16A CBM-54 domains of *Ruminiclostridium thermocellum* F7 Lic16A, *Clostridium themocellum* ATCC 27405 and *Ruminiclostridium thermocellum* DSM 1237 are boxed in red. The protein accession numbers are labeled (gi|995954489|gb|AMJ40768.1| cellulosome-anchoring protein precursor [*Clostridium propionicum* DSM 1682]; gi|312181728|gb|ADQ41898.1| transglutaminase domain-containing protein [*Caldicellulosiruptor kristjanssonii* I77R1B]; gi|311776323|gb|ADQ05809.1| transglutaminase domain protein [*Caldicellulosiruptor hydrothermalis* 108]; gi|311774167|gb|ADQ03654.1| transglutaminase domain-containing protein [*Caldicellulosiruptor owensensis* OL]; gi|302573648|gb|ADL41439.1| transglutaminase domain-containing protein [*Caldicellulosiruptor obsidiansis* OB47]; gi|326543798|gb|ADZ85657.1| Glucan endo-1,3-beta-D-glucosidase [*Clostridium lentocellum* DSM 5427]; gi|145411118|gb|ABP68122.1| S-layer domain protein [*Caldicellulosiruptor saccharolyticus* DSM 8903]; gi|955265344|gb|ALP73406.1| endo-1,3(4)-beta-glucanase, partial [*Caldicellulosiruptor* sp. F32]; gi|312201661|gb|ADQ44988.1| Glucan endo-1,3-beta-D-glucosidase [*Caldicellulosiruptor kronotskyensis* 2002]; gi|302573661|gb|ADL41452.1| Glucan endo-1,3-beta-D-glucosidase [*Caldicellulosiruptor obsidiansis* OB47]; gi|311776336|gb|ADQ05822.1| Glucan endo-1,3-beta-D-glucosidase [*Caldicellulosiruptor hydrothermalis* 108]; gi|373943664|gb|AEY64585.1| Ig-like domain-containing protein, putative S-layer protein [*Clostridium* sp. BNL1100]; gi|219998061|gb|ACL74662.1| S-layer domain protein [*Clostridium cellulolyticum* H10]; gi|961447213|gb|ALS25414.1| SLH domain-containing protein [*Paenibacillus naphthalenovorans*]; gi|1022705355|gb|ANA79717.1| S-layer protein [*Paenibacillus glucanolyticus*]; gi|1031374757|gb|ANF98139.1| hypothetical protein AR543_20420 [*Paenibacillus bovis*]; gi|261280556|gb|ACX62527.1| S-layer domain protein [*Paenibacillus* sp. Y412MC10]; gi|1049752686|gb|ANY65940.1| hypothetical protein BBD42_05270 [*Paenibacillus* sp. BIHB4019]; gi|686546821|gb|AIQ57517.1| hypothetical protein PBOR_11680 [*Paenibacillus borealis*]; gi|686541165|gb|AIQ51862.1| hypothetical protein R70331_10275 [*Paenibacillus* sp. FSL R7-0331]; gi|686535581|gb|AIQ46279.1| hypothetical protein R70723_10605 [*Paenibacillus* sp. FSL R7-0273]; gi|1049752686|gb|ANY65940.1| hypothetical protein BBD42_05270 [*Paenibacillus* sp. BIHB4019]; gi|686541165|gb|AIQ51862.1| hypothetical protein R70331_10275 [*Paenibacillus* sp. FSL R7-0331]; gi|686535581|gb|AIQ46279.1| hypothetical protein R70723_10605 [*Paenibacillus* sp. FSL R7-0273]; gi|1049753427|gb|ANY66681.1| hypothetical protein BBD42_09565 [*Paenibacillus* sp. BIHB4019]; gi|662720239|gb|AIE61449.1| putative secreted protein [*Bacillus methanolicus* MGA3]; gi|375301816|gb|AFA47950.1| hypothetical protein Awo_c11660 [*Acetobacterium woodii* DSM 1030]; gi|375303290|gb|AFA49424.1| hypothetical protein Awo_c26710 [*Acetobacterium woodii* DSM 1030]; gi|375300765|gb|AFA46899.1| S-layer domain containing protein [*Acetobacterium woodii* DSM 1030]; gi|995956117|gb|AMJ42396.1| endoglucanase precursor [*Clostridium propionicum* DSM 1682]; gi|134052896|gb|ABO50867.1| S-layer domain protein [*Desulfotomaculum reducens* MI-1]; gi|974001378|dbj|BAU26536.1| Cellulosome-anchoring protein precursor [*Aneurinibacillus soli*]; gi|686555149|gb|AIQ65844.1| hypothetical protein PSTEL_24790 [*Paenibacillus stellifer*]; gi|686516405|gb|AIQ27106.1| hypothetical protein P40081_02015 [*Paenibacillus* sp. FSL P4-0081]; gi|1028462806|gb|ANE45729.1| hypothetical protein SY83_04795 [*Paenibacillus swuensis*]; gi|605573144|gb|AHV95811.1| S-layer domain protein [*Paenibacillus sabinae* T27]; gi|332696012|gb|AEE90469.1| S-layer domain-containing protein [*Tepidanaerobacter acetatoxydans* Re1]; gi|311777858|gb|ADQ07344.1| glycoside hydrolase family 43 [*Caldicellulosiruptor hydrothermalis* 108]; gi|312201710|gb|ADQ45037.1| glycoside hydrolase family 16 [*Caldicellulosiruptor kronotskyensis* 2002]; gi|312201700|gb|ADQ45027.1| glycoside hydrolase family 16 [*Caldicellulosiruptor kronotskyensis* 2002]; gi|1049073550|gb|ANW97748.1| hypothetical protein CSTERTH_01225 [*Clostridium stercorarium* subsp. thermolacticum DSM 2910]; gi|472398113|gb|AGI38312.1| xylan-binding protein [*Clostridium stercorarium* subsp. stercorarium DSM 8532]; gi|1049073550|gb|ANW97748.1| hypothetical protein CSTERTH_01225 [*Clostridium stercorarium* subsp. thermolacticum DSM 2910]; gi|343963700|gb|AEM72847.1| S-layer domain-containing protein [*Caldicellulosiruptor lactoaceticus* 6A]; gi|312181573|gb|ADQ41743.1| S-layer domain-containing protein [*Caldicellulosiruptor kristjanssonii* I77R1B]; gi|247542485|gb|ACS99503.1| glycoside hydrolase family 16 [*Paenibacillus* sp. JDR-2]; gi|806915496|emb|CQR58104.1| hypothetical protein PRIO_5717 [*Paenibacillus riograndensis* SBR5]; gi|247542612|gb|ACS99630.1| Licheninase [*Paenibacillus* sp. JDR-2]; gi|482514681|gb|AGK07609.1| PglB [*Paenibacillus* sp. S09]; gi|384088723|gb|AFH60159.1| BglA2 [*Paenibacillus mucilaginosus* K02]; gi|336299299|gb|AEI42402.1| BglA2 [*Paenibacillus mucilaginosus* KNP414]; gi|378567862|gb|AFC28172.1| BglA2 [*Paenibacillus mucilaginosus* 3016]; gi|686549654|gb|AIQ60350.1| hypothetical protein PBOR_27925 [*Paenibacillus borealis*]; gi|302777163|gb|ADL67722.1| Mannan endo-1,4-beta-mannosidase [*Thermoanaerobacterium thermosaccharolyticum* DSM 571]; gi|389569310|gb|AFK85715.1| glycoside hydrolase family 26 [*Thermoanaerobacterium saccharolyticum* JW/SL-YS485]; gi|806911027|emb|CQR53170.1| Mannan endo-1,4-beta-mannosidase [*Paenibacillus riograndensis* SBR5]; gi|686552417|gb|AIQ63112.1| hypothetical protein PSTEL_08405 [*Paenibacillus stellifer*]; gi|1049753193|gb|ANY66447.1| beta-mannosidase [*Paenibacillus* sp. BIHB4019]; gi|336297576|gb|AEI40679.1| Mannan endo-1,4-beta-mannosidase [*Paenibacillus mucilaginosus* KNP414]; gi|384090036|gb|AFH61472.1| mannan endo-1,4-beta-mannosidase [*Paenibacillus mucilaginosus* K02]; gi|378568983|gb|AFC29293.1| Mannan endo-1,4-beta-mannosidase [*Paenibacillus mucilaginosus* 3016]; gi|973198192|gb|ALX07409.1| Glucan endo-1,3-beta-D-glucosidase [R*uminiclostridium thermocellum* AD2]; gi|1046311560|gb|ANV75148.1| Glucan endo-1,3-beta-D-glucosidase [*Ruminiclostridium thermocellum* DSM 2360]; gi|316939453|gb|ADU73487.1| Glucan endo-1,3-beta-D-glucosidase [*Ruminiclostridium thermocellum* DSM 1313]; gi|359826770|gb|AEV69543.1| beta-propeller domain-containing protein, methanol dehydrogenase [*Clostridium clariflavum* DSM 19732]; gi|1049078145|gb|ANX02323.1| S-layer protein [*Clostridium stercorarium* subsp. leptospartum DSM 9219]; gi|1049075499|gb|ANW99697.1| S-layer protein [*Clostridium stercorarium* subsp. thermolacticum DSM 2910]; gi|472400210|gb|AGI40409.1| xylan-binding protein [Clostridium stercorarium subsp. stercorarium DSM 8532]; gi|160428582|gb|ABX42145.1| S-layer domain protein [*Lachnoclostridium phytofermentans* ISDg]; gi|373946072|gb|AEY66993.1| Ig-like domain-containing surface protein [*Clostridium* sp. BNL1100].
(TIF)

S1 Table Result of the homology search with the ChiW CBM-54 domain. (PDF)
