# Supplementary figures and images for: Crystal Structure of Chitinase ChiW from Paenibacillus sp. str. FPU-7 Reveals a Novel Type of Bacterial Cell-Surface-Expressed Multi-Modular Enzyme Machinery
Source: PLoS One. 2016 Dec 1;11(12):e0167310. doi: 10.1371/journal.pone.0167310 (PMC5132251; doi:10.1371/journal.pone.0167310)

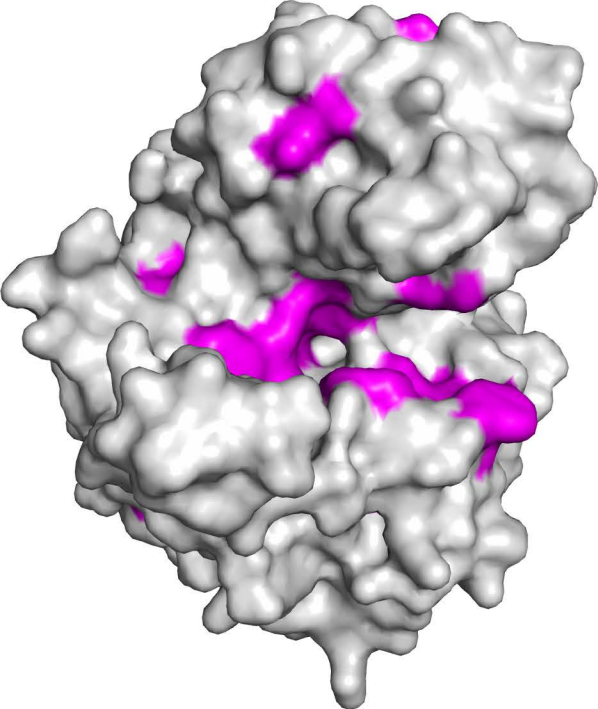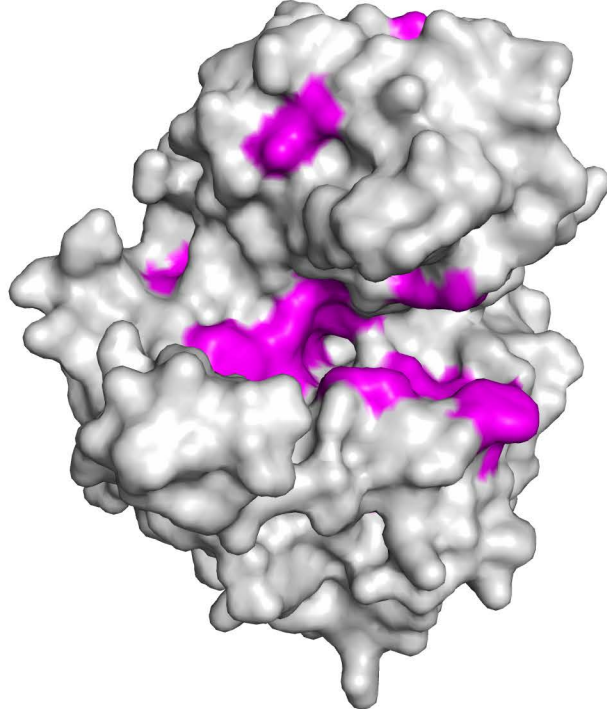

**Cat-1**

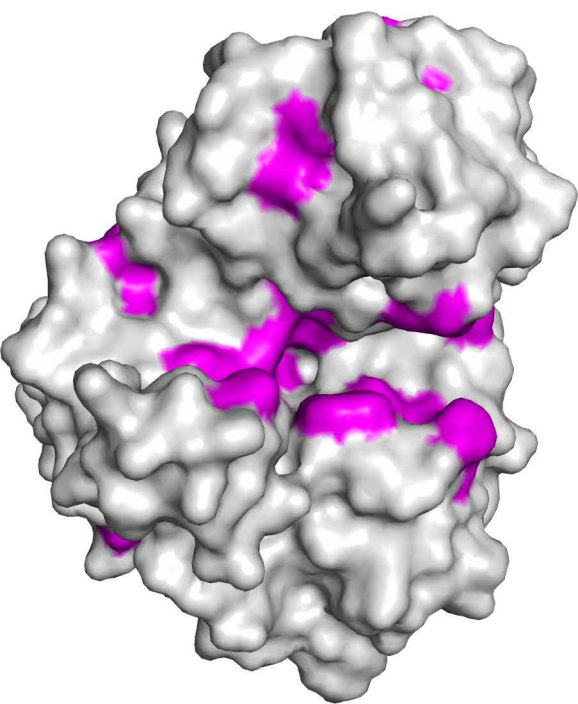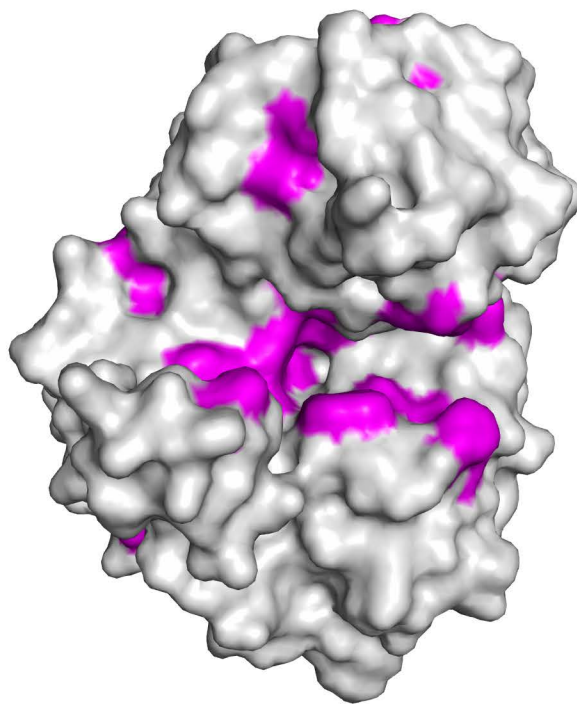

**Cat-2**

Supplement: S1 Fig — The side chains of the aromatic residues (Trp, Phe and Tyr) are shown in magenta. The shapes of the clefts are similar. (PDF) [file pone.0167310.s001.pdf]

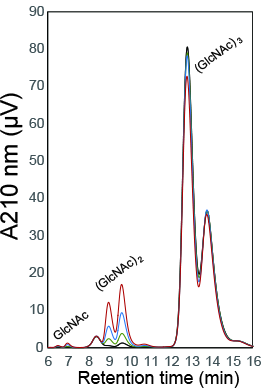

Supplement: S2 Fig — The reaction solution consisted of 5 mM sodium acetate buffer (pH 5.5), 2 mM (GlcNAc)3 and 100 nM ChiW-CD in a 100 μl reaction volume. The reaction was terminated by withdrawing 10 μl aliquots from the reaction solution and then adding 10 μl acetonitrile at 1 (black line), 3 (green line), 10 (blue line) and 20 min (red line). The mixture (5 μl) was subsequently separated on a TSKgel Amide-80 column (4.6 × 250 mm; Tosoh Co., Tokyo, Japan) using 70% (v/v) acetonitrile and detected at 210 nm. The alpha and beta anomers of GlcNAc, (GlcNAc)2 and (GlcNAc)3 were separated by this column under these conditions. (TIF) [file pone.0167310.s002.tif]

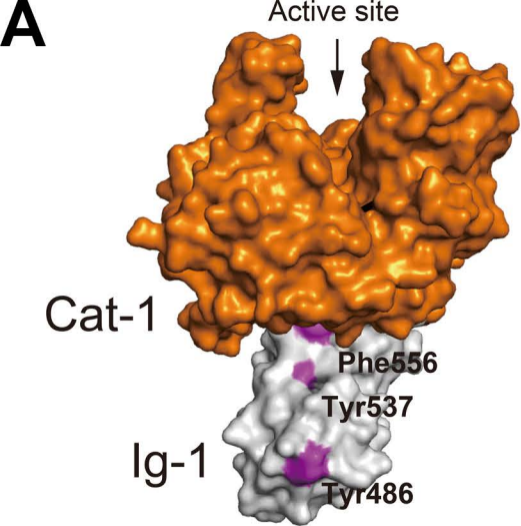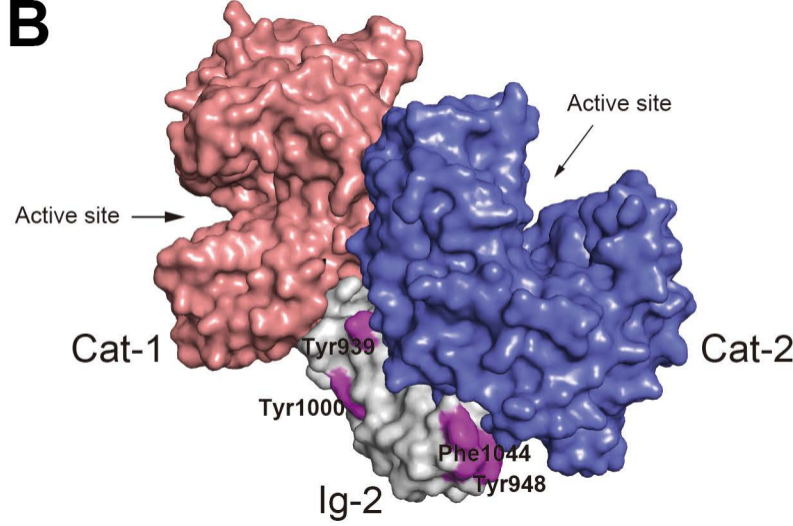

Supplement: S3 Fig — Molecular surface models of Ig-1 and Cat-1 (A) and Cat-1, Ig-2 and Cat-2 (B). Ig-1 and Ig-2 domains are located on the opposite side of the catalytic clefts. The aromatic residues located on the surface of Ig-1 (Tyr486, Tyr537 and Phe556) and surface of Ig-2 (Tyr939, Tyr948, Tyr1000 and Phe1044) are shown in magenta. (PDF) [file pone.0167310.s003.pdf]

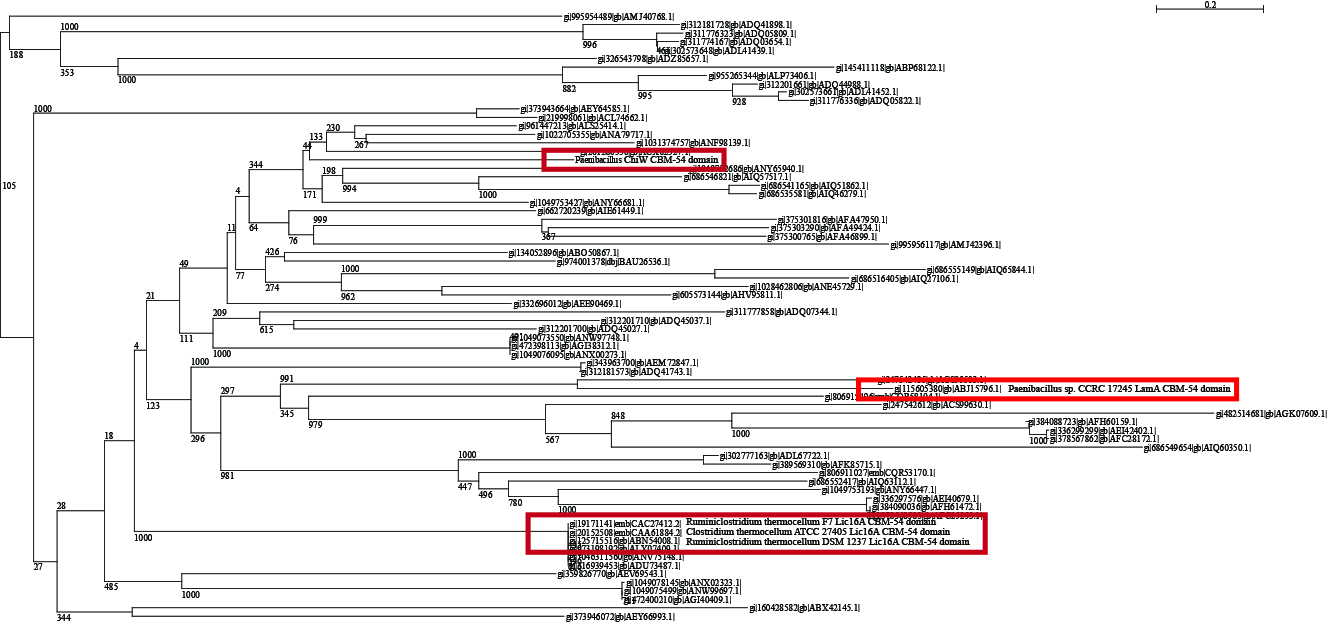

Supplement: S6 Fig — The amino acid sequences of 74 CBM-54 family members were taken from the CAZy database and aligned by ClustalW. The tree is constructed by the neighbor-joining method with 1,000 bootstrap replications. Numbers at branching points refer to bootstrap values. The characterized CBM-54 domains, Paenibacillus ChiW CBM-54 domain, Paenibacillus sp. CCRC 17245 LamA CBM-54 domain, Lic16A CBM-54 domains of Ruminiclostridium thermocellum F7 Lic16A, Clostridium themocellum ATCC 27405 and Ruminiclostridium thermocellum DSM 1237 are boxed in red. The protein accession numbers are labeled (gi|995954489|gb|AMJ40768.1| cellulosome-anchoring protein precursor [Clostridium propionicum DSM 1682]; gi|312181728|gb|ADQ41898.1| transglutaminase domain-containing protein [Caldicellulosiruptor kristjanssonii I77R1B]; gi|311776323|gb|ADQ05809.1| transglutaminase domain protein [Caldicellulosiruptor hydrothermalis 108]; gi|311774167|gb|ADQ03654.1| transglutaminase domain-containing protein [Caldicellulosiruptor owensensis OL]; gi|302573648|gb|ADL41439.1| transglutaminase domain-containing protein [Caldicellulosiruptor obsidiansis OB47]; gi|326543798|gb|ADZ85657.1| Glucan endo-1,3-beta-D-glucosidase [Clostridium lentocellum DSM 5427]; gi|145411118|gb|ABP68122.1| S-layer domain protein [Caldicellulosiruptor saccharolyticus DSM 8903]; gi|955265344|gb|ALP73406.1| endo-1,3(4)-beta-glucanase, partial [Caldicellulosiruptor sp. F32]; gi|312201661|gb|ADQ44988.1| Glucan endo-1,3-beta-D-glucosidase [Caldicellulosiruptor kronotskyensis 2002]; gi|302573661|gb|ADL41452.1| Glucan endo-1,3-beta-D-glucosidase [Caldicellulosiruptor obsidiansis OB47]; gi|311776336|gb|ADQ05822.1| Glucan endo-1,3-beta-D-glucosidase [Caldicellulosiruptor hydrothermalis 108]; gi|373943664|gb|AEY64585.1| Ig-like domain-containing protein, putative S-layer protein [Clostridium sp. BNL1100]; gi|219998061|gb|ACL74662.1| S-layer domain protein [Clostridium cellulolyticum H10]; gi|961447213|gb|ALS25414.1| SLH domain-containing protein [Pae [file pone.0167310.s006.tif]
