# Supplementary material for: Crystal Structure of Chitinase ChiW from Paenibacillus sp. str. FPU-7 Reveals a Novel Type of Bacterial Cell-Surface-Expressed Multi-Modular Enzyme Machinery
Source: PLoS One. 2016 Dec 1;11(12):e0167310. doi: 10.1371/journal.pone.0167310 (PMC5132251; doi:10.1371/journal.pone.0167310)
Supplement: S4 Fig — The numbers in the left-hand column are the protein accession numbers (ZP_07902840: S-layer domain protein [Paenibacillus vortex V453]; YP_004027356: s-layer domain-containing protein [Caldicellulosiruptor kristjanssonii I77R1B]; YP_004797824: S-layer protein [Caldicellulosiruptor lactoaceticus 6A]; ZP_07387876: S-layer domain protein [Paenibacillus curdlanolyticus YK9]; YP_003008964: S-layer protein [Paenibacillus sp. JDR-2]; ZP_08511493: hypothetical protein HMPREF9413_5209 [Paenibacillus sp. HGF7]; EIJ83768: S-layer domain protein [Bacillus methanolicus MGA3]; ZP_09079447: S-layer domain-containing protein [Paenibacillus elgii B69]; YP_003009590: glycoside hydrolase family 16 [Paenibacillus sp. JDR-2]; ABJ15796: endo-beta-1,3-glucanase [Paenibacillus sp. CCRC 17245]; YP_005047467: beta-propeller domain-containing protein, methanol dehydrogenase [Clostridium clariflavum DSM 19732]; ZP_08278755: hypothetical protein HMPREF9412_0339 [Paenibacillus sp. HGF5]; YP_003240334: S-layer domain-containing protein [Paenibacillus sp. Y412MC10]; YP_006468477: cell wall-binding protein [Desulfosporosinus acidiphilus SJ4]; YP_006189237: mannan endo-1,4-beta-mannosidase [Paenibacillus mucilaginosus K02]; YP_004640549: mannan endo-1,4-beta-mannosidase [Paenibacillus mucilaginosus KNP414]; YP_005312442: mannan endo-1,4-beta-mannosidase [Paenibacillus mucilaginosus 3016]; YP_002506677: S-layer protein [Clostridium cellulolyticum H10]; YP_003850806: Mannan endo-1,4-beta-mannosidase [Thermoanaerobacterium thermosaccharolyticum DSM 571]; YP_004310855: glucan endo-1,3-beta-D-glucosidase [Clostridium lentocellum DSM 5427]; YP_006391314: glycoside hydrolase family 26 [Thermoanaerobacterium saccharolyticum JW/SL-YS485]; YP_004459776: S-layer domain-containing protein [Tepidanaerobacter acetatoxydans Re1]; ZP_08191088: S-layer domain-containing protein [Clostridium papyrosolvens DSM 2782]; ZP_09079445: hypothetical protein PelgB_33676 [Paenibacillus elgii B69]; ZP_08420347: putative S-layer [file pone.0167310.s004.pdf]

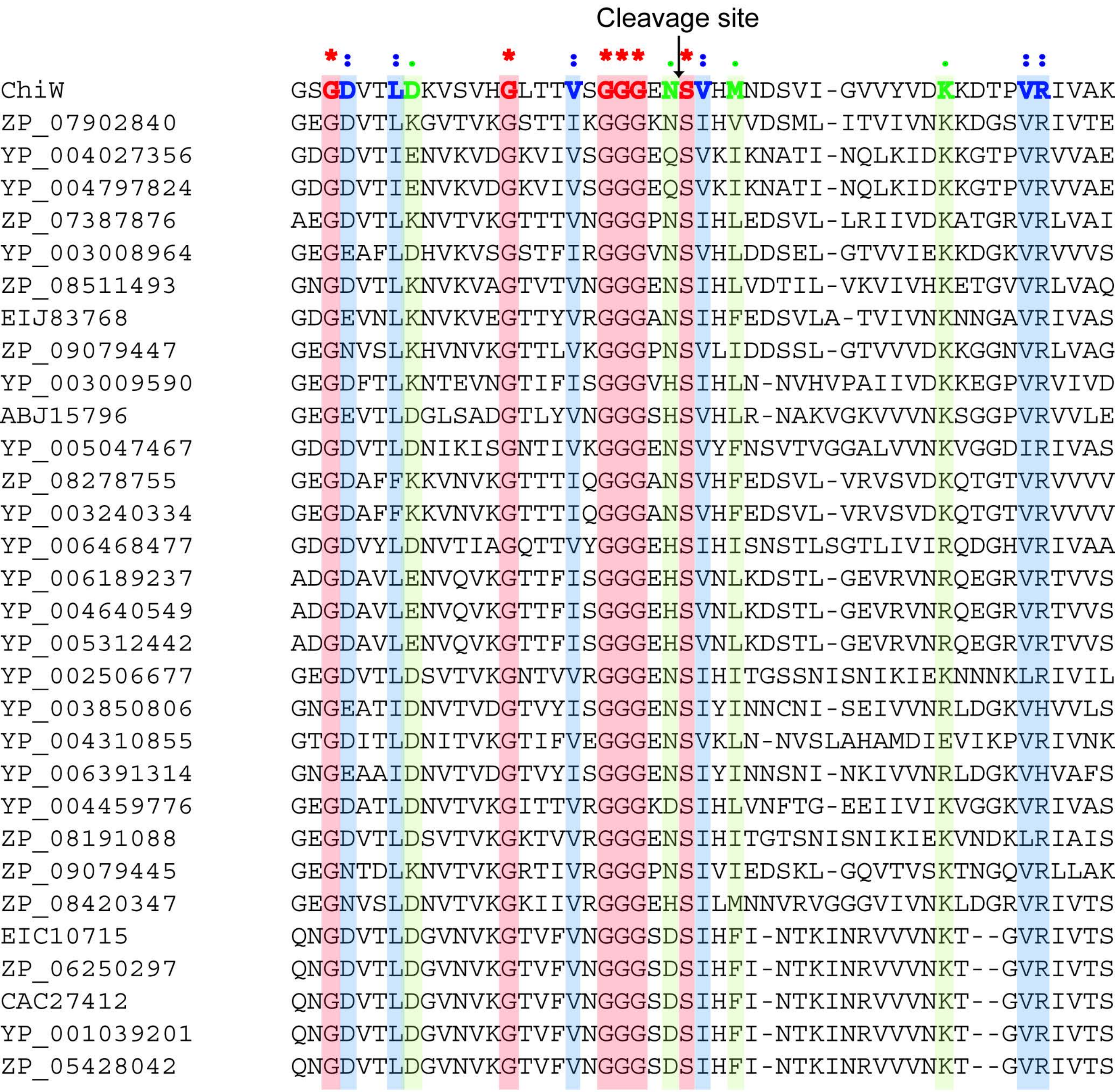
Cleavage site
* : : . * : *** . * : . . : :
ChiW GSGDVTLDKVSVHGLTTVSGGGENSVHMNDSVI-GVVYVDKKDTPVRIVAK
ZP_07902840 GEGDVTLKGVTVKGSTTIKGGGKNSIHVVDSML-ITVIVNKKDGSVRIVTE
YP_004027356 GDGDVTIENVKVDGKVIVSGGGEQSVKIKNATI-NQLKIDKKGTPVRVVAE
YP_004797824 GDGDVTIENVKVDGKVIVSGGGEQSVKIKNATI-NQLKIDKKGTPVRVVAE
ZP_07387876 AEGDVTLKNVTVKGTTTVNGGGPNSIHLEDSVL-LRIIVDKATGRVRLVAI
YP_003008964 GEGEAFLDHVKVSGSTFIRGGGVNSVHLDDSEL-GTVVIEKKDGKVRVVVS
ZP_08511493 GNGDVTLKNVKVAGTVTVNGGGENSIHLVDTIL-VKVIVHKETGVVRLVAQ
EIJ83768 GDGEVNLKNVKVEGTTYVRGGGANSIHFEDSVLA-TVIVNKNNGAVRIVAS
ZP_09079447 GEGNVSLKHVNVKGTTLVKGGGPNSVLIDDSSL-GTVVVDKKGGNVRLVAG
YP_003009590 GEGDFTLKNTEVNGTIFISGGGVHSIHLN-NVHVPAIIVDKKEGPVRVIVD
ABJ15796 GEGEVTLDGLSADGTLYVNGGGSHSVHLR-NAKVGKVVVNKSGGPVRVVLE
YP_005047467 GDGDVTLDNIKISGNTIVKGGGENSVYFNSVTVGGALVVNKVGGDIRIVAS
ZP_08278755 GEGDAFFKKVNVKGTTTIQGGGANSVHFEDSVL-VRVSVDKQTGTVRVVVV
YP_003240334 GEGDAFFKKVNVKGTTTIQGGGANSVHFEDSVL-VRVSVDKQTGTVRVVVV
YP_006468477 GDGDVYLDNVTIAGQTTVYGGGEHSIHISNSTLSGTLIVIRQDGHVRIVAA
YP_006189237 ADGDAVLENVQVKGTTFISGGGEHSVNLKDSTL-GEVRVNRQEGRVRTVVS
YP_004640549 ADGDAVLENVQVKGTTFISGGGEHSVNLKDSTL-GEVRVNRQEGRVRTVVS
YP_005312442 ADGDAVLENVQVKGTTFISGGGEHSVNLKDSTL-GEVRVNRQEGRVRTVVS
YP_002506677 GEGDVTLDSVTVKGNTVVRGGGENSIHITGSSNISNIKIEKNNNKLRIVIL
YP_003850806 GNGEATIDNVTVDGTVYISGGGENSIYINNCNI-SEIVVNRLDGKVHVVLS
YP_004310855 GTGDITLDNITVKGTIFVEGGGENSVKLN-NVSLAHAMDIEVIKPVRIVNK
YP_006391314 GNGEAAIDNVTVDGTVYISGGGENSIYINNSNI-NKIVVNRLDGKVHVAFS
YP_004459776 GEGDATLDNVTVKGITTVRGGGKDSIHLVNFTG-EEIIVIKVGGKVRIVAS
ZP_08191088 GEGDVTLDSVTVKGKTVVRGGGENSIHITGTSNISNIKIEKVNDKLRIAIS
ZP_09079445 GEGNTDLKNVTVKGRTIVRGGGPNSIVIEDSKL-GQVTVSKTNGQVRLLAK
ZP_08420347 GEGNVSLDNVTVKGKIIVRGGGEHSILMNNVRVGGGVIVNKLDGRVRIVTS
EIC10715 QNGDVTLDGVNVKGTVFVNGGGSDSIHFI-NTKINRVVVNKT--GVRIVTS
ZP_06250297 QNGDVTLDGVNVKGTVFVNGGGSDSIHFI-NTKINRVVVNKT--GVRIVTS
CAC27412 QNGDVTLDGVNVKGTVFVNGGGSDSIHFI-NTKINRVVVNKT--GVRIVTS
YP_001039201 QNGDVTLDGVNVKGTVFVNGGGSDSIHFI-NTKINRVVVNKT--GVRIVTS
ZP_05428042 QNGDVTLDGVNVKGTVFVNGGGSDSIHFI-NTKINRVVVNKT--GVRIVTS
