# Supplementary material for: Crystal Structure of Chitinase ChiW from Paenibacillus sp. str. FPU-7 Reveals a Novel Type of Bacterial Cell-Surface-Expressed Multi-Modular Enzyme Machinery
Source: PLoS One. 2016 Dec 1;11(12):e0167310. doi: 10.1371/journal.pone.0167310 (PMC5132251; doi:10.1371/journal.pone.0167310)
Supplement: S5 Fig — ChiW CBM-54, CBM-54 domain of Paenibacillus sp. str. FPU-7 ChiW; Lic16A CBM-54, CBM-54 domain of Ruminiclostridium thermocellum DSM 1237 lichenase A; and LamA CBM-54, CBM-54 domain of Paenibacillus sp. CCRC 17245 endo-β-1,3-glucanase (LamA). Identical amino acid residues among the conserved proteins are indicated by asterisks, whereas colon and period characters indicate conserved residues. The limited proteolysis motif is indicated by a red box. The red and blue characters indicate highly conserved residues. The amino acid residues, Asp262, Ser283, His285 and Arg304 are located at the cleavage site of ChiW. (PDF) [file pone.0167310.s005.pdf]

```
ChiW CBM-54      --------------VILDRAAAFKNEANAIAYDKAGTYGPAS------------------
Lic16A CBM-54    -NYTSKFKDGSLVKEYAKDSVSALVEKGYIAGYEDGTFRPDNYITRAETIKILNKIIPSL
LamA CBM-54      AAVLSGFKDRTAIGGFAVQPLADLVSAGALKGFADGTLRPQQPLTRAEAVVLLDRLAGEI
                                    . :   . . :     **  * .

                                                            Asp262
ChiW CBM-54      -------GTETIDGNVKVTVPGVTLRNLVIKGDLLLSEGVGSGDVTLDKVSVHGLTTVSG
Lic16A CBM-54    YNEKGDYKNEEVAGNALINTEGVILKDTVINGDLYLAQGIQNGDVTLDGVNVKGTVFVNG
LamA CBM-54      IRQPGSYDGVKSDSGLLIASADTILKQAEVKGNVLITAGVGEGEVTLDGLSADGTLYVNG
                              ..  :   .. *::  ::*:: :: *: .*:**** :...*   *.*
                                                         Limited proteolysis motif

                     Ser283
                       His285                Arg304
ChiW CBM-54      GGENSVHMNDSVIGVVYVDKKDTPVRIVAKGSAKVGEVIIAGSVKLEETDLTGTG-----
Lic16A CBM-54    GGSDSIHFINTKINRVVVNKTG--VRIVTSGNTSVESVVVKSGAKLEEKELTGDG-----
LamA CBM-54      GGSHSVHLRNAKVGKVVVNKSGGPVRVVLEGSSKVGEMSLETGAVVEVGEQAEVASLQVE
                 **..*:*: :: :. * *:*..  **:* .*.:.* .: :  .. :*  : :  .

ChiW CBM-54      ---------FEKVVLKDLLPANAKVTLSGSFTDVDVAASANPQLNVNSGTIERLTVAASS
Lic16A CBM-54    ---------FKNVTVDSQLSAGNEIIFVGDFEQVDVLADDALLETKEAKMKLRIFGQRIK
LamA CBM-54      QSAGGTELNVKGTVGELQTQASGVTLNGETFEQGKVLEVQQGKAADKTEPQNGNAPAGGT
                          .: .. .    *.        * : .*          ::           .

ChiW CBM-54      KD----------------------------------------------------------
Lic16A CBM-54    VNGKAIEKSS--------------------------------------------------
LamA CBM-54      SGGGAASPGNGGGSGGGGNGGGGTAGENLAAPVLTPDPVNNVLGRDVALTFADNPAWRNA
                  .

ChiW CBM-54      --------AVIVLASGVKVTTLTLNIKTQIKGQGSVGTAVVNLGGKGSSFESAPGKTEGI
Lic16A CBM-54    ---------KNYIVNGELISTEEEPGPSDAPGAEDDQNSGSPGSSTNPAPTKNPNEEWRL
LamA CBM-54      ISEITLNGRKLTLTADYLLSAGSLTLKASVFAETGDHTLIIKAAGYTDVSVTQPMGKWEL
                             :. .  :::      :.  .  .  .     ..      . *     :

ChiW CBM-54      AKDSVTTGGSF
Lic16A CBM-54    VWSDEFNGS--
LamA CBM-54      VWGDEFDGSGT
                 . ..   *.
```
