# Supplementary material for: Crystal Structure of Chitinase ChiW from Paenibacillus sp. str. FPU-7 Reveals a Novel Type of Bacterial Cell-Surface-Expressed Multi-Modular Enzyme Machinery
Source: PLoS One. 2016 Dec 1;11(12):e0167310. doi: 10.1371/journal.pone.0167310 (PMC5132251; doi:10.1371/journal.pone.0167310)
Supplement: S1 Table — (PDF) [file pone.0167310.s007.pdf]

S1 Table. Result of homology search with the ChiW CBM-54 domain.

Accession Number	Source organism	Description	CBM-54/total amino acid	No. of SLH	Enzyme components	Score	E value	Identity (%)
ZP_07387876	*Paenibacillus curdlanolyticus*	S-Layer domain protein	195-444/862	3		224	2.00E-64	46
ZP_08511493	*Paenibacillus* sp.	Hypothetical protein	202-437/1670	3	GH-18 chitinase x 3	219	2.00E-61	47
ZP_07902840	*Paenibacillus vortex*	S-Layer domain protein	205-436/1106	3		205	5.00E-57	44
ZP_08278755	*Paenibacillus* sp.	Hypothetical protein	198-441/896	3		199	1.00E-55	43
YP_003240334	*Paenibacillus* sp.	S-Layer domain-containing protein	198-441/900	3		199	2.00E-55	42
YP_001113692	*Desulfotomaculum reducens*	S-Layer domain-containing protein	195-441/1081	3		176	1.00E-46	42
ZP_09640026	*Desulfitobacterium dichloroeliminans*	Cell wall binding repeat 2-containing protein	351-576/1464			156	9.00E-40	42
YP_003008964	*Paenibacillus* sp.	S-Layer protein	236-448/4640	3		155	3.00E-39	39
EIJ83768	*Bacillus methanolicus*	S-Layer domain protein	292-507/1348			152	2.00E-38	38
YP_004022846	*Caldicellulosiruptor kronotskyensis*	Glycoside hydrolase family 16	207-410/2435	3	GH-16 β-glucanase	145	2.00E-35	37
ZP_09079445	*Paenibacillus elgii*	Hypothetical protein	203-467/1556	3	Endo nuclease	144	2.00E-35	36
YP_004459776	*Tepidanaerobacter acetatoxydans*	S-Layer domain-containing protein	210-426/782	3		142	4.00E-35	41
ZP_09079447	*Paenibacillus elgii*	S-Layer domain-containing protein	197-479/1735	3	GH-16 β-glucanase	138	3.00E-33	36

YP_004022856	*Caldicellulosiruptor kronotskyensis*	Glycoside hydrolase family 16	208-442/2229	3	GH-16 β-glucanase	130	2.00E-30	33
YP_005267788	*Acetobacterium woodii*	S-Layer domain containing protein	247-451/1453			121	1.00E-27	37
YP_005047467	*Clostridium clariflavum*	beta-Propeller domain-containing protein	188-379/1001	3		119	4.00E-27	35
YP_005270313	*Acetobacterium woodii*	Hypothetical protein	248-459/1266			115	1.00E-25	35
EIC10715	*Ruminiclostridium thermocellum*	Glycoside hydrolase family 16	203-344/1255	3	GH-16 β-glucanase	114	4.00E-25	41
ZP_06250297	*Ruminiclostridium thermocellum*	Glycoside hydrolase family 16	203-344/1321	3	GH-16 β-glucanase	114	4.00E-25	41
CAC27412.2	*Ruminiclostridium thermocellum*	endo-1,3(4)-beta-Glucanase	203-344/1321	3	GH-16 β-glucanase	114	4.00E-25	41
YP_001039201	*Ruminiclostridium thermocellum*	Glycoside hydrolase family protein	203-344/1321	3	GH-16 β-glucanase	114	4.00E-25	41
ZP_05428042	*Ruminiclostridium thermocellum*	Glycoside hydrolase family 16	203-344/1468	3	GH-16 β-glucanase	114	5.00E-25	41
YP_006468477	*Desulfosporosinus acidiphilus*	Cell wall-binding protein	369-642/975			113	5.00E-25	30
ZP_03734647	*Dethiobacter alkaliphilus*	Ig domain protein group 2 domain protein	42-243/517			107	2.00E-23	32
YP_005268839	*Acetobacterium woodii*	Hypothetical protein	251-456/1459			105	2.00E-22	31
YP_004027356	*Caldicellulosiruptor kristjanssonii*	S-Layer domain-containing protein	188-440/1495	3		103	1.00E-21	32
ZP_08420347	*Ruminococcaceae bacterium*	Putative S-layer homology domain protein	222-392/1004	3		101	4.00E-21	36
YP_004797824	*Caldicellulosiruptor lactoaceticus*	S-Layer protein	188-440/480	3		99.4	7.00E-21	32

YP_002506677	*Clostridium cellulolyticum*	S-Layer protein	207-409/1421	3		99.4	4.00E-20	35
YP_005311321	*Paenibacillus mucilaginosus*	Hypothetical protein	217-407/1611	3	GH-16 β-glucanase	98.6	6.00E-20	35
YP_004642272	*Paenibacillus mucilaginosus*	Hypothetical protein	217-407/1611	3	GH-16 β-glucanase	98.6	6.00E-20	35
YP_006187924	*Paenibacillus mucilaginosus*	Hypothetical protein	139-299/1520	2	GH-16 β-glucanase	98.6	6.00E-20	38
ZP_08191088	*Clostridium papyrosolvens*	S-Layer protein	207-437/1428	3		97.4	1.00E-19	33
YP_003850806	*Thermoanaerobacterium thermosaccharolyticum*	Mannan endo-1,4-beta-mannosidase	220-381/1410	3	GH-26 endo-1,4-beta-mannosidase	95.5	6.00E-19	31
YP_004266737	*Syntrophobotulus glycolicus*	Cell wall binding repeat 2-containing protein	339-532/554			94	7.00E-19	30
YP_001558884	*Clostridium phytofermentans*	S-Layer protein	204-413/2117	1		95.1	9.00E-19	30
YP_004310855	*Clostridium lentocellum*	Glucan endo-1,3-beta-D-glucosidase	213-392/1683	3	GH-16 β-glucanase	93.6	2.00E-18	33
ZP_07388838	*Caldicellulosiruptor hydrothermalis*	Glucan endo-1,3-beta-D-glucosidase	196-402/2048	3	GH-16 β-glucanase	93.2	4.00E-18	32
YP_003992713	*Caldicellulosiruptor hydrothermalis*	Glycoside hydrolase family 43	189-346/1440	1	GH-43 β-xylosidase	92.8	4.00E-18	35
YP_006391314	*Thermoanaerobacterium saccharolyticum*	Glycoside hydrolase family protein	229-381/1422	3	GH-26 endo-1,4-beta-mannosidase	92	8.00E-18	31
YP_752867	*Syntrophomonas wolfei*	Hypothetical protein	200-322/758	3		90.9	1.00E-17	42
YP_005148798	*Clostridium* sp.	Ig-like domain-containing surface protein	207-409/1331	2		90.5	2.00E-17	31
